# Vitamin D receptor promotes healthy microbial metabolites and microbiome

**DOI:** 10.1038/s41598-020-64226-7

**Published:** 2020-04-30

**Authors:** Ishita Chatterjee, Rong Lu, Yongguo Zhang, Jilei Zhang, Yang Dai, Yinglin Xia, Jun Sun

**Affiliations:** 1 Division of Gastroenterology and Hepatology, Department of Medicine, University of Illinois at Chicago, Chicago, USA; 2 Department of Bioengineering, University of Illinois at Chicago, Chicago, USA

**Keywords:** Transcription, Metabolic syndrome, Gastrointestinal diseases

## Abstract

Microbiota derived metabolites act as chemical messengers that elicit a profound impact on host physiology. Vitamin D receptor (VDR) is a key genetic factor for shaping the host microbiome. However, it remains unclear how microbial metabolites are altered in the absence of VDR. We investigated metabolites from mice with tissue-specific deletion of VDR in intestinal epithelial cells or myeloid cells. Conditional VDR deletion severely changed metabolites specifically produced from carbohydrate, protein, lipid, and bile acid metabolism. Eighty-four out of 765 biochemicals were significantly altered due to the *Vdr* status, and 530 significant changes were due to the high-fat diet intervention. The impact of diet was more prominent due to loss of VDR as indicated by the differences in metabolites generated from energy expenditure, tri-carboxylic acid cycle, tocopherol, polyamine metabolism, and bile acids. The effect of HFD was more pronounced in female mice after VDR deletion. Interestingly, the expression levels of farnesoid X receptor in liver and intestine were significantly increased after intestinal epithelial VDR deletion and were further increased by the high-fat diet. Our study highlights the gender differences, tissue specificity, and potential gut-liver-microbiome axis mediated by VDR that might trigger downstream metabolic disorders.

## Introduction

Metabolites are the language between microbiome and host^1^. To understand how host factors modulate the microbiome and consequently alter molecular and physiological processes, we need to understand the metabolome — the collection of interacting metabolites from the microbiome and host.

Vitamin D/VDR signaling contributes to the genetic, environmental, immune, and microbial aspects of human diseases (e.g., inflammatory bowel disease and obesity)^2,3^. The human *Vdr* gene is the first gene identified as a vital host factor that shapes the gut microbiome at the genetic level^4^. In mice lacking VDR, we observed significant shifts in the microbiota relative to control mice. In humans, correlations between the microbiota and serum measurements of selected bile acids and fatty acids were detected^4^. Those metabolites include known ligands and downstream metabolites of VDR^5^. Moreover, we have demonstrated that VDR knock out (KO) (*Vdr*^−/−^) mice have depleted *Lactobacillus* and enriched *Clostridium* and *Bacteroides* in feces. Notably, in the cecal content, *Alistipes* and *Odoribacter* were significantly reduced whereas *Eggerthella* was increased^6^. Intestinal specific deletion of VDR (VDR^ΔIEC^) leads to microbial dysbiosis due to a decrease in the butyrate-producing bacteria^7,8^. However, it is unclear how the loss of VDR impacts microbial metabolites.

In the current study, we hypothesize that host factors (e.g., VDR status in specific tissues) modulate microbial metabolites and the microbiome, thus contributing to the high risk of metabolic diseases. We used intestinal epithelium-specific VDR knock out (VDR^ΔIEC^) mice and myeloid cell-specific VDR KO (VDR^Δlyz^) mice to assess whether the microbiome-associated metabolic changes linked with conditional loss of VDR in a particular tissue. Because the majority of metabolic syndromes are multifactorial, we further evaluated the effect of high-fat diet (HFD) on VDR^ΔIEC^ mice as compared to control chow diet-fed mice. We also correlated the altered metabolite profiles to specific mechanisms that lead to the observed changes in the host and microbiome.

## Results

### Deletion of intestinal epithelial VDR impacted the overall metabolite profile

First, we examined the effects of intestinal epithelial VDR on the metabolite profile. Among named biochemical compounds, VDR^ΔIEC^ mice exhibited alterations in 68 metabolites (of which 35 increased and 33 decreased) with P ≤ 0.05 significance level and 55 biochemicals with 0.05 < P < 0.1 significance level (of which 25 increased and 30 decreased) (Table 1).

**Table 1:** Intestinal epithelial VDR on the profile of metabolites.

Statistical Comparisons		
ANOVA contrasts	VDR^ΔIEC^/VDR^LoxP^	
	Chow	
Total biochemicals p ≤ 0.05	68	
Biochemicals (↑↓)	35	33
Total biochemicals 0.05 < p < 0.10	55	
Biochemicals (↑↓)	25	30

**Figure 1:**
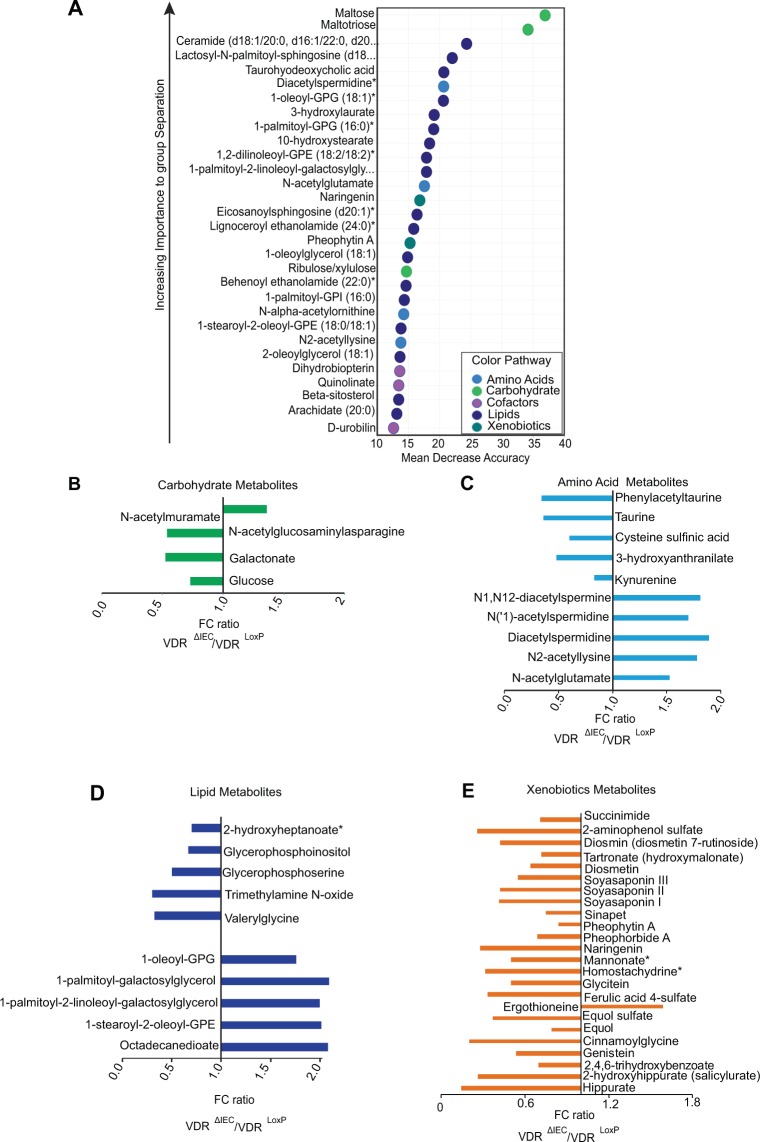
Impact of intestinal epithelial VDR deletion on metabolite profile: (**A**) Random Forest (RF) analysis showing the top 30 most important metabolites resulting from using biochemical data derived from VDR^LoxP^, VDR^∆IEC^ and VDR^∆lyz^ mice fecal samples. The variables are ordered top-to-bottom as most-to-least important in categorizing between VDR deleted (VDR^∆IEC^ and VDR^∆lyz)^ and VDR^LoxP^ groups. Different color indicates specific metabolites resulting from different super-pathways like light blue = amino acid; green =  carbohydrate, purple = cofactors, blue = lipids, Teal= Xenobiotics. Fold change (FC) ratios of the average concentrations of metabolites between VDR^ΔIEC^ mice to that in the VDR^LoxP^. Graph denotes only those biochemicals which displayed maximum fold change. Metabolites are listed as their origin of metabolic pathways: (**B**) carbohydrate (**C**) amino acid (**D**) lipid and (**E**) xenobiotic. Differences are assessed by the Mann–Whitney U test. VDR^ΔIEC^ (N  =  17) & VDR^LoxP^ (N  =  16) mice. Significance is established at adjusted 0.05 < P < 0.1.

Random Forest (RF) analysis of metabolites among chow diet-fed animals revealed the impact of different metabolites. Figure 1A shows a list of the top 30 biochemicals that contribute to maximum importance. Of these, maltose, maltotriose, and ceremide are among the top three most significant differentially expressed biochemicals. The action of the intestinal microbiome is responsible for the generation of several metabolites derived from carbohydrates, amino acids, bile acids, heme, and other dietary sources. Several of these metabolites are reabsorbed in the gut and can bind to cellular receptors, thus potentially influencing host functions^9^. The most significant alterations in metabolites were generated by four main super-pathways including (A) carbohydrate, (B) protein/ amino acids, (C) lipid, and (D) xenobiotics metabolism.

#### Carbohydrate metabolism

Carbohydrates are the primary source of energy for gut microbiota. Colonic bacteria ferment nondigestible complex-carbohydrates and release glycogen, amino-sugars, and pentoses^10^. These are considered major factors in shaping the composition and physiology of the microbiome. As shown in Fig. 1B, VDR^ΔIEC^ had a significant (P ≤ 0.05) increase in amino-sugar metabolite N-acetylmuramate compared to the VDR^LoxP^ control mice. Conversely, N-acetylglucosaminylasparagine, glucose, and galactonate were downregulated in the VDR^ΔIEC^ group (Fig. 1B). These results suggest that the loss of intestinal epithelial VDR in host modifies the carbohydrate metabolism, thus affecting mainly glycolysis, gluconeogenesis, pyruvate, fructose, mannose galactose, and amino-sugar metabolism pathways.

#### Protein/Amino-acid metabolism

Gut bacteria produce a range of metabolites by synthesizing proteinogenic amino acids via protein fermentation^11^. These metabolites are known to exert beneficial or harmful effects on the host. VDR^ΔIEC^ mice showed increased levels of N1,N12-diacetylspermine, N(‘1)-acetylspermidine, N-acetylglutamate, N2-acetyllysine, and diacetylspermidine **(**Fig. 1C), indicating a significant surge in polyamine, glutamate, and lysine metabolism. A decrease in tryptophan and methionine metabolism in the VDR^ΔIEC^ group was indicated by decreased taurine, kynurine, and other related metabolites (Fig. 1C).

#### Lipid metabolism

Gut bacteria affect lipid metabolism through multiple direct and/or indirect mechanisms, including bile acid metabolism, cholesterol transport, and energy expenditure^12,13^. We found a significant increase of octadecanedioate, 1-stearoyl-2-oleoyl-GPE, 1-palmitoyl-2-linoleoyl-galactosylglycerol, 1-palmitoyl-galactosylglycerol, and 1-oleoyl-GPG in VDR^ΔIEC^ mice compared to those in the VDR^LoxP^ mice. In contrast, the levels of valerylglycine, trimethylamine N-oxide, glycerophosphoserine, glycerophosphoinositol, and 2-hydroxyheptanoate were decreased in the VDR^ΔIEC^ mice (Fig. 1D), indicating that fatty acid and phospholipid metabolism was altered in the VDR^ΔIEC^ mice.

#### Xenobiotics/Others

Xenobiotics are the extrinsic molecules ingested by the host from the environment and are subsequently metabolized by microorganisms and transformed into hundreds of metabolites^14^. VDR deficiency in the intestinal epithelium altered the levels of many xenobiotics. RF analysis of animals showed that naringenin, a flavonoid displays strong anti-inflammatory and antioxidant function, among the pool of top 30 metabolites (Fig. 1A). Further ANOVA analysis indicates that most xenobiotics were significantly downregulated in the VDR^ΔIEC^ mice (Fig. 1E). Ergothioneine (ET), an anti-oxidant sulfur-containing derivative of the histidine (amino acid) was the only xenobiotic found to be upregulated in VDR^IEC^ (Fig. 1E).

### Myeloid cell-specific VDR knockdown contributes to the definitive alteration of metabolites indicating the tissue-specific role of host VDR

VDR is known to have tissue and cell-specific roles^15^. Hence, we examined our myeloid cell-specific VDR KO model. In the VDR^Δlyz^ mice, 100 known biochemicals were found to be significantly altered with P-value ≤0.05 (of which 56 increased and 44 decreased) and 58 chemicals showed with significance level 0.05 < P < 0.10 (of which 28 increased and 30 decreased) (Table 2), compared to chow-fed VDR^LoxP^ control animals.

**Table 2:** Myeloid cell-specific VDR contributes to the alteration of metabolites different from the Intestinal epithelial VDR.

Statistical Comparison						
Welch’s Two-Sample *t*-Test	Chow					
	VDR^ΔLYZ^/VDR^LoxP^			VDR^ΔLYZ^/VDR^ΔIEC^		
	All	Female	Male	All	Female	Male
Total biochemicals p ≤ 0.05	100	74	147	118	44	122
Biochemicals (↑↓)	56|44	52|22	46|101	59|59	26|18	43|79
Total biochemicals 0.05 < p < 0.10	58	45	105	49	31	64
Biochemicals (↑↓)	28|30	24|21	20|85	26|23	19|12	15|49

We compared the change in metabolites derived from VDR^ΔIEC^ and VDR^Δlyz^ mice by Welch’s two-sample *t*-test and found 118 metabolites were significantly changed (P ≤ 0.05) (black box, Table 2) and another 49 fell in the significant level 0.05 < P < 0.1. Here, we focused on the major changes in the carbohydrate, amino acids, lipid, and xenobiotics metabolites in VDR^∆lyz^ model.

**Figure 2:**
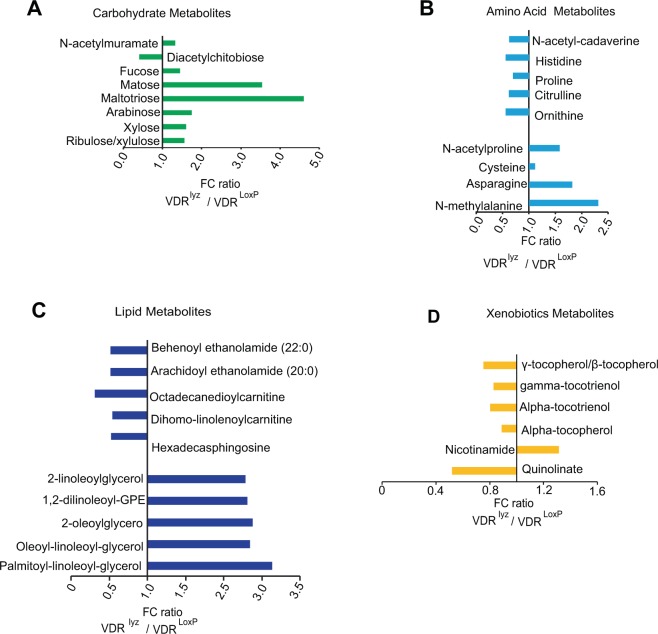
Overall alteration in the metabolites following myeloid cell-specific VDR deletion: Fold change ratio generated by (**A**) carbohydrate (**B**) amino acids (**C**) lipid and (**D**) xenobiotics metabolism in VDR^∆lyz^ mice. The graph represents only those biochemicals showing maximum alterations among known detectable metabolites. Differences are assessed by the Mann–Whitney U test. VDR^∆lyz^ (N  =  10) & VDR^LoxP^ (N  =  16). Significance is established at adjusted 0.05 < P < 0.1.

Loss of VDR in myeloid cells in the VDR^∆lyz^ group showed altered carbohydrate metabolism and increased levels of the amino sugar metabolite N-acetylmuramate (Fig. 2A), similar to the VDR^ΔIEC^ mice. Unlike the VDR^ΔIEC^ group, VDR^∆lyz^ mice showed an increase in pentose and glycogen metabolism in conjunction with elevated amounts of ribulose/xylulose, xylose, arabinose, maltotriose, maltose, and fucose (Fig. 2A). A prominent decrease in diacetylchitobiose in VDR^Δlyz^ mice was observed. The VDR^∆lyz^ group also showed decreased histidine, proline, and citrulline, which was accompanied by a rise in N-acetyl proline and asparagine levels, compared to VDR^LoxP^ (Fig. 2B). These data suggest a reduction in urea-arginine-proline metabolism and elevated alanine and aspartate metabolism in VDR^∆lyz^ mice.

In contrast to VDR^LoxP^ mice, VDR^∆lyz^ mice demonstrated a significant increase in palmitoyl-linoleoyl-glycerol and a substantial decrease in sphingosines and fatty acid metabolism, indicated by metabolites like hexadecasphingosine and dihomo-linolenoylcarnitine (Fig. 2C). Remarkably, among the different xenobiotic metabolism pathways, the VDR^Δlyz^ group displayed a significant downregulation of quinolinate and tocopherol pathway derived metabolites and an increase in nicotinamide (Fig. 2D). Thus, these data indicate the different roles of VDR in intestinal epithelial cells and myeloid cells.

### VDR deletion significantly impacted bile acid profile, especially in females

**Figure 3:**
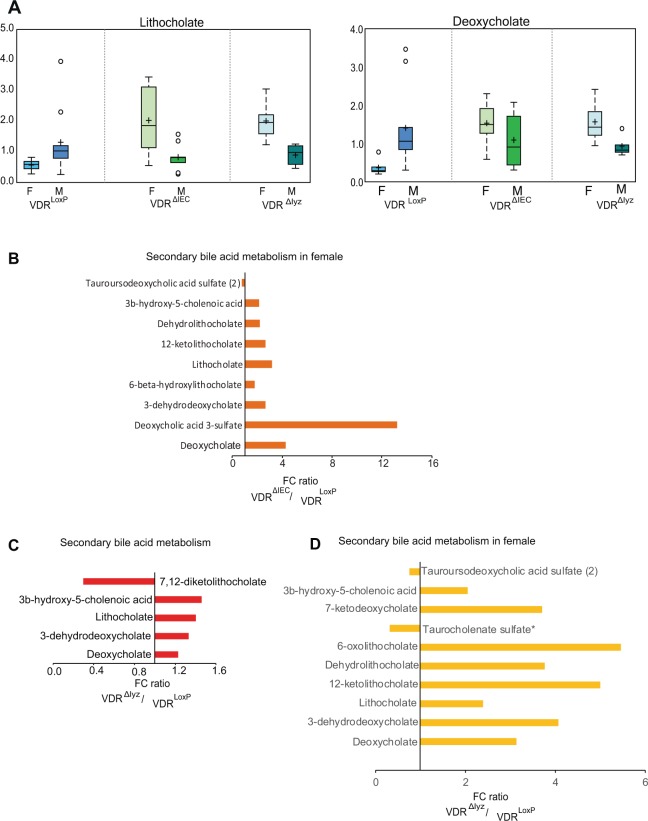
VDR deletion altered bile acid (BA) metabolism: Box-plot diagrams displaying changes in secondary bile acid (**A**) lithocholate and deoxycholate in control VDR^LoxP^ group as compared to VDR^ΔIEC^ and VDR^∆lyz^ mice. (**B**) Specific changes in secondary bile acid metabolites as noted in female VDR^∆IEC^ mice. (**C**) Collective changes demonstrated by VDR^∆lyz^ mice. (**D**) Definite variations in secondary bile acid metabolites displayed by female VDR^∆lyz^ mice. The data presented as the fold change (FC) ratios of the average concentrations of identified BA species in respective groups. VDR^ΔIEC^ group (N =  17; F = 8, M = 9), VDR^∆lyz^ (N  =  10; F = 5, M = 5) & VDR^LoxP^ (N  =  16; F = 6, M = 10). Differences are assessed by the Mann–Whitney U test. Significance is established at an adjusted 0.05 < P < 0.1.

VDR is known to function as a bile acid sensor in the intestine and loss of VDR is known to disquiet the bile acid homeostasis^16,17^. Here, we assessed modifications in metabolites from secondary bile acid metabolism pathways. The microbiota converts primary bile acids to secondary bile acids, which are then reabsorbed and can affect diverse biological processes^18^. Among different secondary bile acids, lithocholate, and deoxycholate, were increased due to loss of VDR in VDR^ΔIEC^ and in VDR^∆lyz^ mice (Fig. 3A). When comparing VDR^ΔIEC^ females to VDR^LoxP^ females, we found an increase in deoxycholic acid 3 sulphate and in deoxycholate (Fig. 3B). A decrease in 7,12-diketolithocholate, was specifically observed in VDR^∆lyz^ female mice (Fig. 3C).

Both VDR^∆IEC^ and VDR^∆lyz^ female mice showed a significant increase in deoxycholate, 3-dehydrodeoxychlate, lithocholate, 12-ketolithocholate, de-hydrolithocholate, and 3b-hydroxy-5-cholenoic acid, compared to control females (Fig. 3B,D). Tauroursodeoxycholic acid sulfate was decreased in the VDR^∆IEC^ and VDR^∆lyz^ female mice. Overall, our results indicate that VDR deletion significantly influences bile acid metabolism in a gender-specific manner.

### VDR deficiency resulted in significant alterations in the polyamines levels

**Figure 4:**
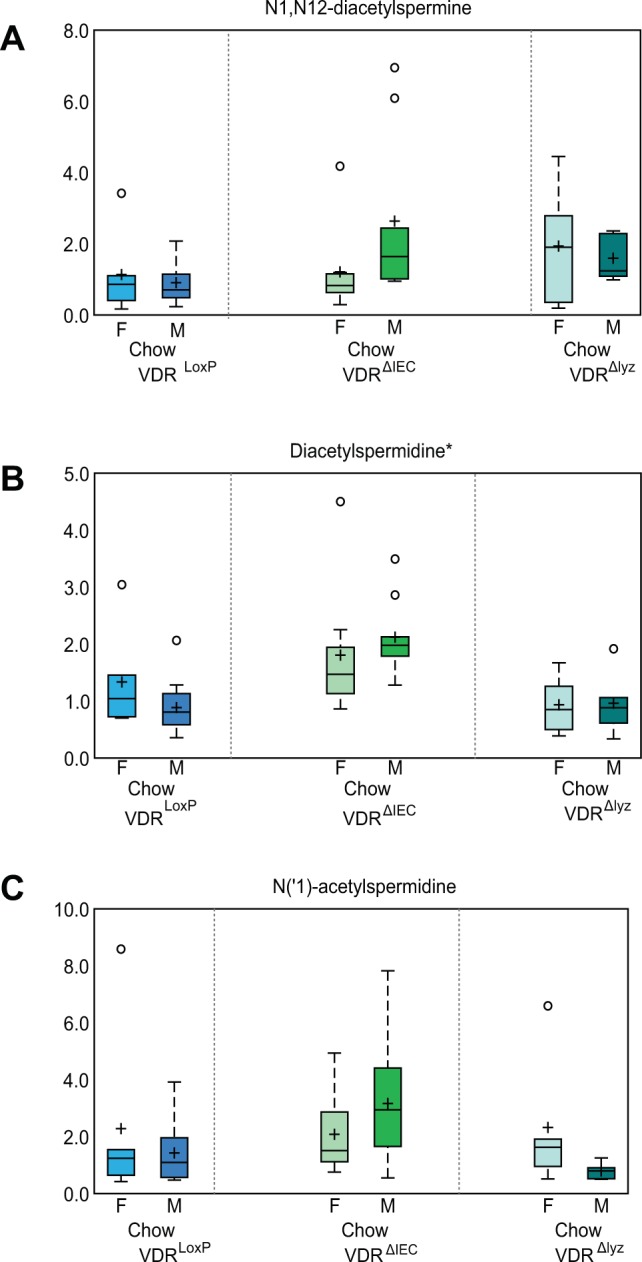
VDR deficiency results in significant alterations in the levels of polyamines: Box-plot diagrams showing increased levels of polyamines metabolites namely, (**A**) N1, N12-diacetylspermine, (**B**) diacetylspermidine, (**C**) N (‘1) acetylspermidine were noted following VDR deletion (in VDR^ΔIEC^ & VDR^∆lyz^) in mice. This data is represented as the BOX-Plot diagram showing maximum and minimum variation among the group. VDR^ΔIEC^ group (N =  17; F = 8, M = 9), VDR^∆lyz^ (N  =  10; F = 5, M = 5) & VDR^LoxP^ (N  =  16; F = 6, M = 10). The ratio of fold-change differences are assessed by the Mann–Whitney U test. Significance is established at adjusted 0.05 < P < 0.1.

Polyamines have been shown to play a role in facilitating a switch between different coactivator complexes that bind to nuclear receptors such as VDR^19^. Here, we observed a significant elevation in polyamines, such as N1, N12-diacetylspermine (Fig. 4A), diacetylspermidine (Fig. 4B), and N (‘1)- acetylspermidine (Fig. 4C) in chow-fed VDR^ΔIEC^ animals, compared with the VDR^LoxP^ mice indicating accumulation of polyamines in VDR deficient animals. As noted, increases in polyamine levels were observed in male VDR^ΔIEC^ mice.

### Long-chain fatty acids (LCFAS) and acylcarnitines were significantly elevated in VDR deficient mice indicating perturbations with β-oxidation

**Figure 5:**
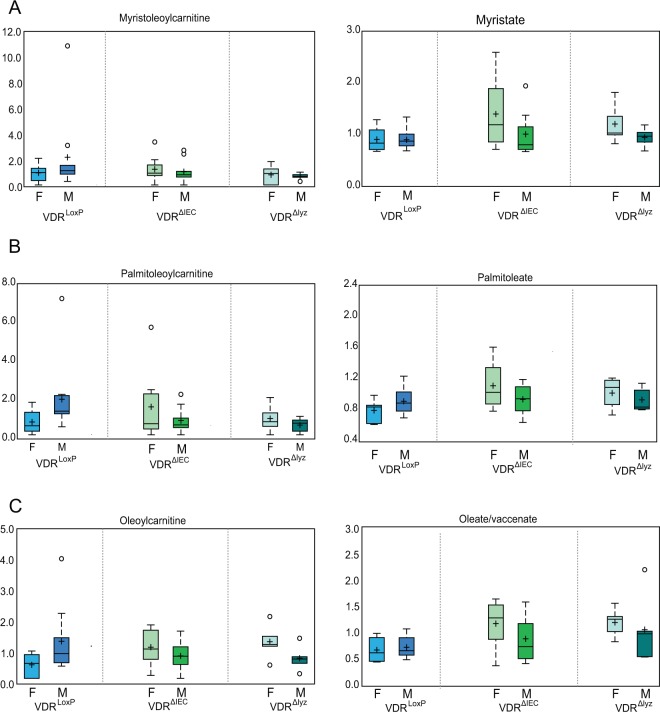
VDR deficiency in mice increased long-chain fatty acids and acylcarnitines: Fecal samples derived from VDR^ΔIEC^ & VDR^∆lyz^ animals showed increased levels of carnitines (**A**) myristoylcarnitine, (**B**) palmitoylcarnitine, (**C**) oleoylcarnitine, as compared to controls. This surge was accompanied by elevated levels of long-chain fatty acids (LCFAs) (**A**) myristate, (**B**) palmitoleate, (**C**) oleate. This data is represented as BOX-Plot diagram showing maximum and minimum variation among the group. This data is represented as BOX-Plot diagram showing maximum and minimum variation among the group. VDR^ΔIEC^ group (N =  17; F = 8, M = 9), VDR^∆lyz^ (N  =  10; F = 5, M = 5) & VDR^LoxP^ (N  =  16; F = 6, M = 10). Significance is established at adjusted 0.05 < P < 0.1.

Fatty acid beta-oxidation is one of the main energy-yielding metabolic processes. An earlier study has shown that Vitamin D/VDR plays an important role in the composition of fatty acids via direct regulation of *Elovl3* (an FA elongase enzyme) expression^20^. Hence, we evaluated the effect of VDR deletion on fatty acid metabolites. We found that carnitines were significantly elevated in fecal samples from VDR^ΔIEC^ and VDR^∆lyz^ mice, compared to the VDR^LoxP^, including myristoylcarnitine (C14), palmitoylcarnitine (C16), oleoylcarnitine (C18:1) **(**Fig. 5A–C**)**. This increase is accompanied by an elevation in long-chain fatty acids **(**Fig. 5A–C**)** that were mostly observed in VDR^ΔIEC^ and VDR^∆lyz^ females, compared to VDR^LoxP^ mice (Table 3). Defects in the beta-oxidation of fatty acids can be evaluated based on acylcarnitines (AC). Substantial increase in acylcarnitines and long-chain fatty acids could be potential indicators of elevated beta-oxidation in VDR deficient animals. However, there is no significant change in 3-hydroxybutyrate (BHBA).

**Table 3:** Long-chain fatty acids and acylcarnitines elevated in VDR deficient mice.

Sub Pathway	Biochemical Name	Fold of Change								
		ANOVA Contrasts						Welch’s 2-Sample t-Test		
		VDR∆^IEC^VDR LoxP^LoxP^		VDR∆^IEC^VDR ^LoxP^				Chow		
								VDR^lyz^ VDR ^LoxP^		
		Chow	HFD	Chow		HFD				
				Female	Male	Female	Male	All	Female	Male
Long Chain Saturated Fatty Acid	myristate (14:0)	1.24	1.23	1.44	1.07	1.52	1	1.19	1.33	1.05
	pentadecanoate (15:0)	1.33	0.87	1.81	0.98	1.07	0.71	1.39	1.97	0.96
	palmitate (16:0)	1.19	1.41	1.33	1.07	2.28	0.87	1.46	1.49	1.42
	margarate (17:0)	1.36	1.5	1.81	1.02	2.69	0.83	1.57	2.26	1.07
	stearate (18:0)	1.15	1.48	1.31	1	2.5	0.88	1.25	1.42	1.12
	nonadecanoate (19:0)	1.3	1.56	1.83	0.92	2.78	0.88	1.27	1.76	0.95
	arachidate (20:0)	1.31	1.7	1.72	1	3	0.96	1.28	1.48	1.14
Long Chain Monounsaturated Fatty Acid	palmitoleate (16:1n7)	1.19	1.05	1.38	1.02	1.06	1.04	1.12	1.28	1.02
	10-heptadecenoate (17:1n7)	1.3	1.09	1.51	1.11	1.24	0.95	1.3	1.59	1.07
	oleate/vaccenate (18:1)	1.37	1.19	1.66	1.13	1.92	0.73	1.59	1.76	1.45
	10-nonadecenoate (19:1n9)	1.32	1.4	1.68	1.03	2.13	0.92	1.65	2.06	1.39
	eicosenoate (20:1)	1.51	1.6	2.23	1.02	2.67	0.96	1.32	1.82	1.08
	erucate (22:1n9)	1.56	1.7	2.74	0.88	2.73	1.05	0.87	1.48	0.66
Fatty Acid Metabolism (Acyl Carnitine, Long Chain Saturated)	myristoylcarnitine (C14)	0.91	2.89	1.34	0.63	1.71	4.9	0.53	1.08	0.33
	palmitoylcarnitine (C16)	1.15	2.6	2.11	0.63	1.9	3.56	1.06	2.34	0.67
	margaroylcarnitine (C17)*	1.03	1.93	1.56	0.68	1.5	2.48	0.94	1.67	0.65
	arachidoylcarnitine (C20)*	1.18	1	1.5	0.93	0.7	1.42	0.83	1.3	0.61
	behenoylcarnitine (C22)*	1.2	0.84	1.53	0.93	0.54	1.32	0.9	1.3	0.72
	lignoceroylcarnitine (C24)*	1.12	0.94	1.4	0.89	0.73	1.21	1.06	1.35	0.91
Fatty Acid Metabolism (Acyl Carnitine, Monounsaturated)	myristoleoylcarnitine (C14:1)*	0.86	2.77	1.28	0.57	3.27	2.35	0.47	0.86	0.35
	palmitoleoylcarnitine (C16:1)*	0.81	2.7	1.47	0.45	2.16	3.37	0.55	1.2	0.35
	oleoylcarnitine (C18:1)	1.18	2.49	2.1	0.66	1.78	3.49	1.01	2.21	0.6
	eicosenoylcarnitine (C20:1)*	1.45	1.74	2.22	0.94	1.23	2.45	1.06	1.98	0.66
Fatty Acid Metabolism (Acyl Carnitine, Polyunsaturated) (Acyl Carnitine, Hydroxy)	linoleoylcarnitine (C18:2)*	0.92	2.1	1.71	0.5	1.44	3.07	0.69	1.64	0.43
	linolenoylcarnitine (C18:3)*	1.61	1.02	2.92	0.89	0.63	1.65	1.25	2.92	0.72
	dihomo-linoleoylcarnitine (C20:2)*	1.15	1.62	1.42	0.94	1.04	2.53	0.78	1.32	0.52
	arachidonoylcarnitine (C20:4)	0.97	1.39	2.52	0.37	0.55	3.53	0.49	1.47	0.21
	(S)-3-hydroxybutyrylcarnitine	1.59	0.49	3.33	0.76	0.37	0.65	1.35	2.79	0.84

### HFD intervention altered metabolite profile in VDR^LoxP^ and VDR^ΔIEC^ mice

**Figure 6:**
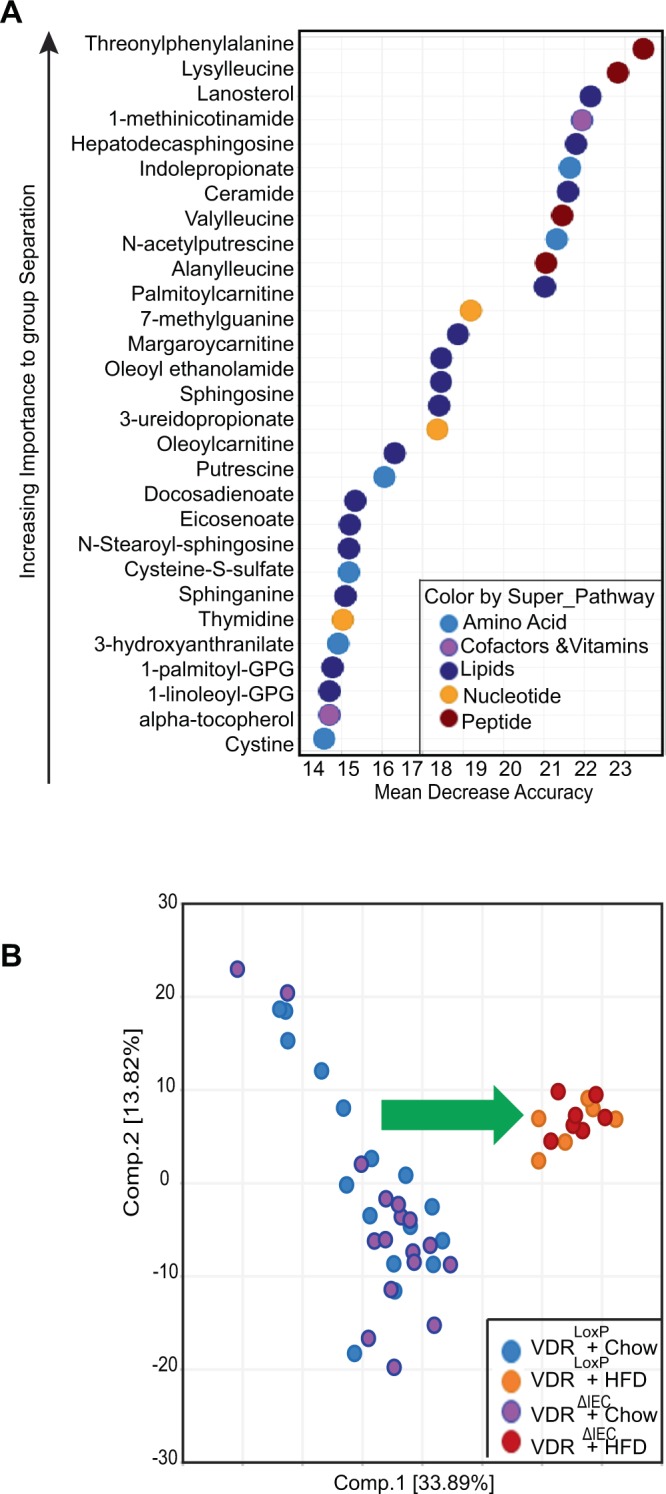
Alteration in the metabolites following VDR Deletion and HFD intervention: (**A**) Random forest (RF) analysis showing the top 30 metabolites responsible for classification as mean decrease accuracy values. Most vital biochemicals are listed from data derived from fecal samples of VDR^LoxP^, VDR^∆IEC^ mice fed with HFD or chow diet. Different colors indicate different biochemicals like, light blue = amino acid, purple = co-factors &vitamins, blue = lipids, orange = nucleotide, red = peptide. (**B**) Principal Component Analysis (PCA) on metabolite level Correlations. Color code: light blue= VDR^LoxP^ + chow, orange = VDR^LoxP^ + HFD, purple= VDR^∆IEC^ chow, red = VDR^∆IEC^ + HFD. VDR^ΔIEC^ group (Chow diet: N =  17; HFD: N = 7), & VDR^LoxP^ (Chow Diet: N  =  16; HFD: N = 6).

Studies have shown that obese humans and mice have microbiomes very different from their lean controls^21–24^. We further evaluated how diet impacted the metabolites in mice with tissue-specific VDR deletion. The 30-top ranking biochemicals in the importance plot suggests key differences in peptides, lipid metabolism, cofactors, vitamins, and amino acids with maximum impact on threonyl phenylalanine (Fig. 6A). RF-classification using named metabolites detected in VDR^LoxP^ and VDR^ΔIEC^ with HFD gave a predictive accuracy of 100%.

**Figure 7:**
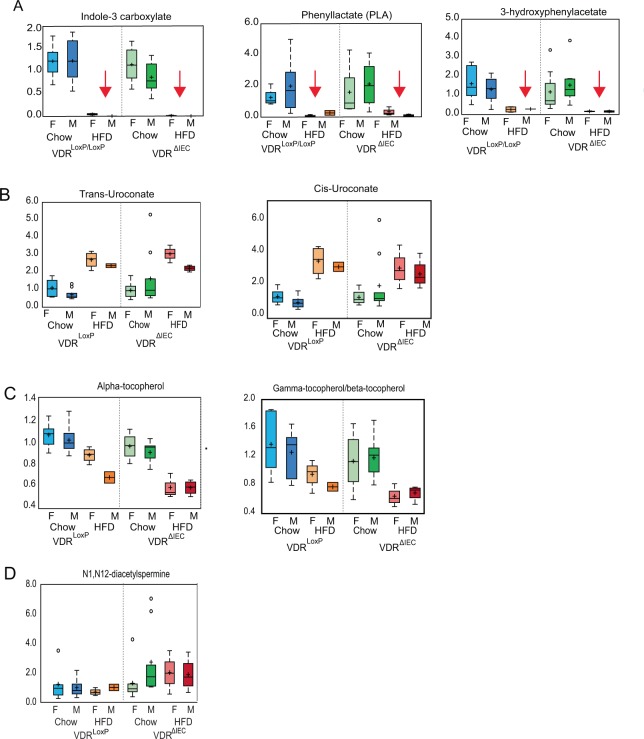
HFD altered the metabolite profile: Intestinal specific VDR deletion along with HFD diet decreased (red arrow) the levels of (**A**) indole 3 carboxylate, PLA, 3-hydroxyphenylacetate and increase in (**B**) trans and cis-uroconate. Altered expression of biochemicals resulting from (**C**) tocopherol and (**D**) polyamine metabolism after HFD feeding. These data are represented as the BOX-Plot diagram showing maximum and minimum variation among the group. VDR^ΔIEC^ group (Chow diet: N =  17; HFD: N = 7) & VDR^LoxP^ (Chow Diet: N  =  16; HFD:N = 6). Significance is established at adjusted 0.05 < P < 0.1.

Principal Component Analysis (PCA) for fecal samples showed clear separation based on the diet (Fig. 6B). Microbiome-derived metabolites had divergent trends following HFD feeding. Aromatic amino acids like phenyl lactate (PLA), phenethylamine, 3-hydroxyphenylacetate, indole 3-carboxylate, indolelactate, and indolepropionate were decreased in both VDR^LoxP^ and VDR^ΔIEC^ HFD groups, as compared to the regular chow diet (Fig. 7A). Alternatively, trans and cis-urocanate were significantly increased by HFD (Fig. 7B). Levels of equol, a microbiota-derived metabolite known to exert epigenetic changes by inhibiting DNA methylation, histone modification, and regulating ncRNAs, was also found to be altered following VDR deletion and HFD.

### Lower levels of tocopherol metabolism were associated with HFD intervention

Interestingly, HFD-fed VDR^ΔIEC^ mice had lower levels of alpha-tocopherol **(**Fig. 7C) in contrast to VDR^LoxP^ and was noted important in RF analysis **(**Fig. 6A). Additional decreases were observed in levels of alpha-tocopherol and gamma tocopherol/betatocopherol (Fig. 7C) in HFD fed animals. Lower levels of tocopherols in HFD fed VDR deficient animals might be indicative of increased risk for colon cancer. Alternatively, polyamines levels in HFD fed VDR^ΔIEC^ animals were also impacted greatly. Here, we observed significant elevation in levels of polyamines, such as spermidine, N1, N2-diacetylspermine, in all VDR^ΔIEC^ animals especially after HFD feeding, as compared to VDR^LoxP^ group (Fig. 7D**)**. These data suggest an accumulation of polyamines in HFD fed VDR deficient animals. Because polyamines have strong anti-inflammatory functions^25^, these changes may impact aspects of host immunity.

### Gender-specific changes in HFD fed mice following VDR deletion

When metabolites were analyzed based on gender, there was a separation between males and females that were fed HFD (Supplementary Fig. 1A,B), but in animals fed chow diet this effect was less significant **(**Fig. 6B**)**.

**Figure 8:**
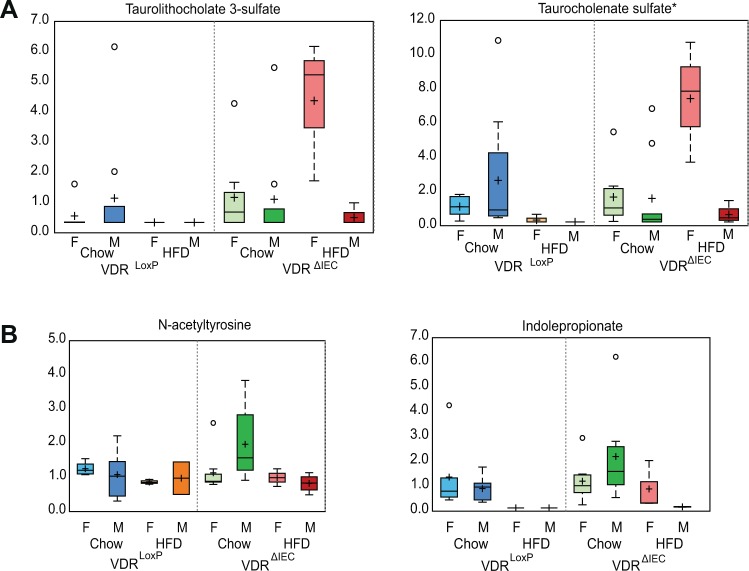
Gender-specific alteration in metabolites displayed following intestinal VDR deletion and HFD intervention. HFD fed female VDR^ΔIEC^ mice showed increased (**A**) taurolithocholate 3-sulphate and taurocholenate sulphate. Variations in the biochemicals namely (**B**) N-acetyltyrosine and indolepropionate. Data are expressed as Boxplot diagram. Differences were assessed by the Mann–Whitney U test. VDR^ΔIEC^ group (Chow diet: N =  17; HFD: N = 7), & VDR^LoxP^ (Chow Diet: N  =  16; HFD: N = 6). Significance is established at adjusted 0.05 < P < 0.1.

Intestinal VDR deficiency extensively alters primary and secondary bile acid metabolites and bile acids are known to shape the gut microbiome especially in obesity. As anticipated, VDR deficiency along with HFD immensely altered the bile acid levels in fecal samples. Specifically, levels of the bile acids, taurolithocholate 3 sulphate, and taurocholenate sulphate were raised in VDR deficient animals (Fig. 8A). Metabolites like N-acetyltyrosine, N-formylphenylalanine, and indolepropionate were significantly changed in the VDR^ΔIEC^, compared to VDR^LoxP^ males; they remain unaltered after HFD feeding, suggesting that diet might impact changes that resulted from VDR deficiency in males (Fig. 8B).

### Metabolites and microbiome regulated by VDR

**Figure 9:**
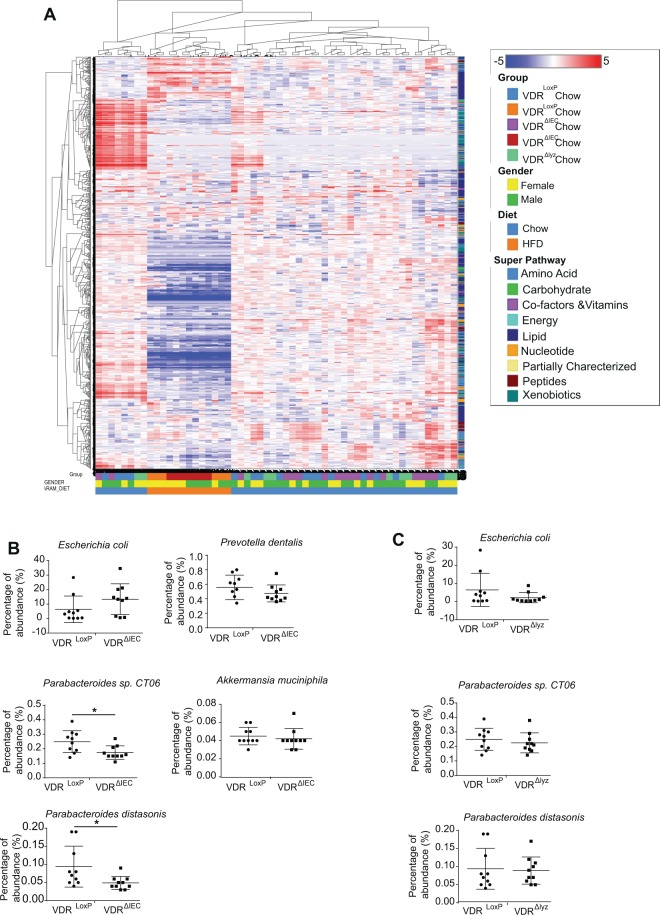
(**A**) Heat map of a global metabolomic study comparing fecal samples from VDR deficient animals to the control group as well as that were kept on HFD or a chow diet. Different group, gender, diet, super-pathways are indicated by colors as indicated in the right panel. Variations in explicit microbial content following targeted VDR deletion in intestine in (**B**) VDR^ΔIEC^ mice and (**C**) myeloid cell-specific VDR^Δlyz^ mice. Each dot indicates percentage of abundance in each mice sample. Significance is established at adjusted P  <  0.05.

Using Hierarchical clustering analysis (HCA) (Fig. 9A), a stepwise clustering method that groups metabolically similar samples close to one another, we found fecal samples did not show primary clustering by genotype (VDR^LoxP^, VDR^ΔIEC^, and VDR^∆lyz^). Because the deletion of intestinal epithelial cell-specific VDR impacted metabolites differently than myeloid cell-specific VDR deletion, we further checked whether these specific metabolite profiles are linked to changes in the microbiome.

Microbial analysis showed VDR^LoxP^ fecal samples contain *Lactobacillus, Butyricimonas, Lactococcus*, while VDR^ΔIEC^ samples contain *Clostridium, Eubacterium, Bacteroides, Tannerella*, and *Prevotella* taxa. The difference in the microbial communities might be related to differences in tryptophan, polyamine, and tocopherol metabolism observed in this study. The abundance of *Parabacteroides* affected by VDR signaling in both human and mouse samples are reported to alter secondary bile acids in obesity^4^. Interestingly, we found dysregulation of secondary bile acids after VDR deletion followed by HFD intervention.

VDR^ΔIEC^ mice showed an increasing trend in *E. coli*, and a decreasing trend of *Prevotella dentalis* and *A. Muciniphila* populations compared to VDR^LoxP^ mice (Fig. 9B). A significant decrease in *Parabacteroides sp. CT06* and *Parabacteroides distasonis* were noted in VDR^ΔIEC^ mice. However, VDR^Δlyz^ mice did not show similar changes (Fig. 9C). Alterations in maltose metabolism in VDR deficient mice (Fig. 1A) could be related to the abundance of *E. coli* in those mice, as shown in our previous studies^7,8^.

### Changes in VDR and FXR in liver and colon with or without HFD

**Figure 10:**
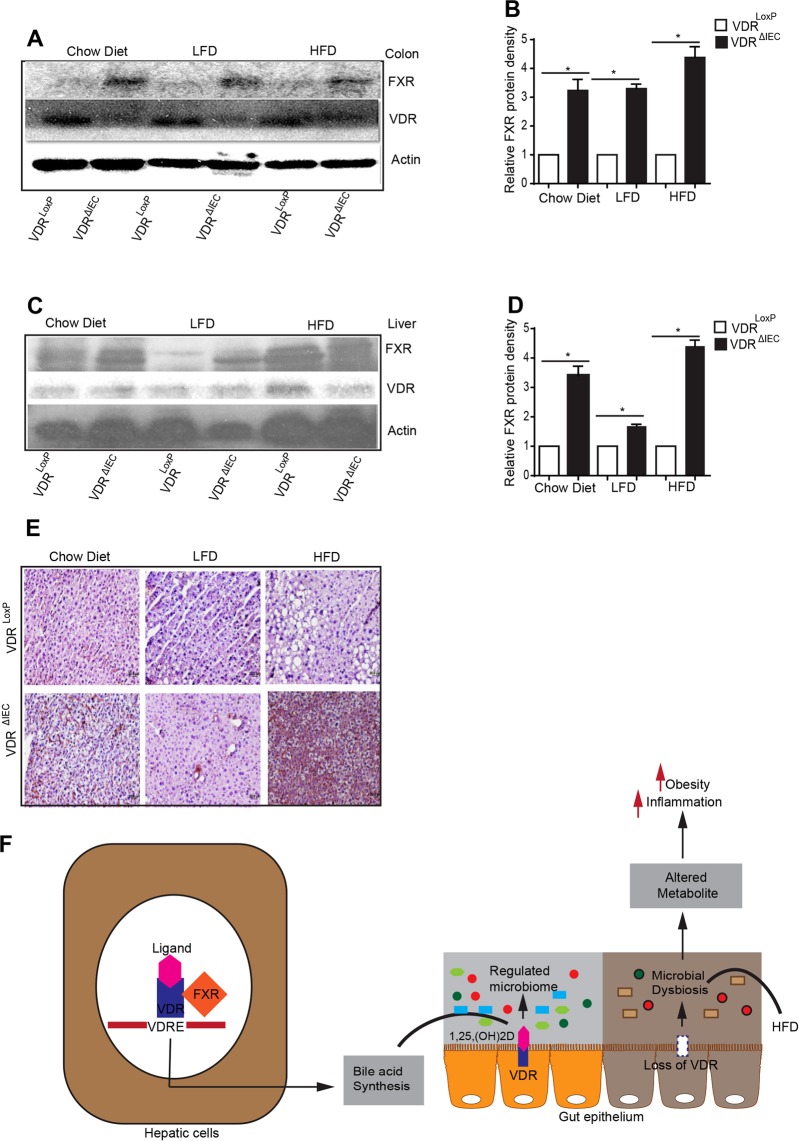
Western blot results showing increased FXR expression in the colonic epithelium (**A**) as well as in liver hepatic cells (**B**) following VDR deletion and HFD. (**C**) IHC staining of FXR (in brown color) in hepatic sections indicated increased expression (N = 3–6). (**D**) A working model showing role of VDR in regulating microbiome and metabolite and obesity. The absence of VDR leads to altered metabolites, which contribute to the disease state. Significance was established at adjusted P  <  0.05.

Two nuclear receptors, VDR and farnesoid X receptor (FXR) interact with each other in a Vitamin D3-independent manner^26^. To verify the changes of VDR and related pathways in addition to microbiome and metabolites, we investigated the protein expression of VDR and FXR in mice with or without HFD. Western blot analysis of FXR indicated a ~ 4-fold and ~5-fold increase in FXR in the colon (Fig. 10A,B) and liver (Fig. 10C,D) of HFD fed VDR^ΔIEC^ mice, respectively. Our metabolite analysis indicated that VDR status significantly impacts bile acid metabolism. Hence, we wanted to check whether hepatic FXR was altered. Immunohistochemical staining (IHC) of liver sections showed a similar increase in FXR, consistent with the observation by western blots.

## Discussion

In the current study, we have demonstrated that targeted deletion of VDR in intestinal or myeloid cells distinctively transformed metabolite profiles and the gut microbiome, leading to an increased risk of obesity. We found that 84 identifiable biochemicals that were significantly altered due to the VDR status. When challenged with a HFD, 530 metabolites showed discrete changes. These changes were observed due to variations in carbohydrate, protein, lipid, and xenobiotic pathways. The deletion of VDR mainly impacted bile acid, LCFA, polyamine, and tocopherol metabolism. VDR^ΔIEC^ mice challenged with HFD diet had the most dramatic changes in metabolites generated from energy expenditure, TCA cycle, tocopherol, and polyamine metabolism. Interestingly, HFD along with loss of VDR influenced female mice more than the males. At the protein level, we found that FXR expression was increased after VDR deletion and that HFD further elevated FXR in the colon and liver of VDR^ΔIEC^ mice. It is known that microbiota-mediated changes in bile acid profiles signal through FXR. FXR contributes directly to diet-induced obesity by promoting increased adiposity and altering the microbiota composition^27^. A study on HFD fed rats showed an increased expression of FXR^28^. Our results clearly indicate that the gut microbiota actively participates in host metabolism by regulating metabolites generated from bile acid metabolism via VDR-FXR signaling. VDR deficiency alters the metabolite profile and FXR expression in the host. Our data suggest the tissue specificity and gender differences of VDR in regulating metabolites and impacting gut-liver axis (Fig. 10F).

Our study highlights the importance of carbohydrate, lipid, and amino acid metabolism following VDR deletion indicating changes in glycogen metabolism as well as lipid and amino acid super pathways, whereas RF analysis using data derived from HFD fed VDR^loxP^ and VDR^∆IEC^ mice pointed to peptides, lipid metabolism, cofactors, vitamins (e.g., alpha-tocopherol), and amino acid metabolism. Loss of intestinal VDR increased taurine and kynurenine levels. The amino acid metabolite taurine is known to be protective against inflammation, apoptosis, and oxidative stress^29^. The kynurenine pathway is associated with inflammatory neurological disorders^30^.

Microbial action in the gut is responsible for the generation of several metabolites derived from bile acids, amino acids, heme, and dietary sources. Bile acids are reabsorbed in the intestine by enterohepatic recirculation and can affect diverse biological processes^31^. In our study, deletion of VDR altered secondary bile acids, signifying the crucial role of VDR in assembling the bile acid pool. Alterations in the bile acid profile were more obvious in females with VDR deletion, as well as in those receiving HFD. Previous studies have shown that the deletion of VDR alters metabolic responses in female mice^32,33^. *Vdr* gene polymorphisms are associated with PCOS and osteoporosis^34^. Bile acids are also considered significant factors in shaping the microbiome of diet-induced obese mice^35^. The dysfunction of the VDR-associated bile acid pathway observed in our study further explains the risk of HFD-induced obesity without the protection of Vitamin D/VDR.

Excessive accumulation of lipids such as long chain acylcarnitines (LCACs), ceramides, and other metabolites are implicated in cell stress and inflammation. Our study demonstrates that long-chain fatty acids (LCFAs) and acylcarnitines are significantly elevated in VDR deficient animals, potentially due to perturbations in β-oxidation. In the absence of vitamin, polyunsaturated fats can be oxidized in the intestines to produce mutagens and subsequently, inflammatory cells in close proximity to the colon can produce reactive oxygen species^36^. As a result, VDR deficiency may cause lower levels of tocopherols, which may be indicative of an increased risk for colon cancer.

VDR plays an important role in regulating several physiological functions through host-microbiome interactions^4^. It is crucial for inflammation and immune responses^15,37,38^. Dysbiosis has emerged as a key risk factor for developing a myriad of metabolic diseases including obesity, atherosclerosis, cardiovascular disease, and Type 2 diabetes^39–41^. A shift in the metabolic capacitance of the microbiota is also associated with the severity of nonalcoholic fatty liver disease (NAFLD)^42^. Our previous studies have shown that VDR knock out (VDR^−/−^) mice have depleted Lactobacillus and enriched *Clostridium* and *Bacteroides* in feces. Notably, in the cecal content, *Alistipes* and *Odoribacter* population were significantly down, and *Eggerthella* were increased. Intestinal specific deletion of VDR leads to dysbiosis due to increased *E. coli* and *Bacteroides* and decreased butyrate-producing bacteria^7^. This imbalance resulted in defective autophagy in colitis^8^. In the current study, we found that VDR deletion contributes to the variation of microbial contents, supporting the changes in metabolite profile. Accordingly, the percentage of abundance of *Parabacteroides distasonis* (PD) significantly dropped in VDR^ΔIEC^ mice. For example, PD is known to modulate host metabolism via FXR pathway by producing secondary bile acids^43^. N-acetylmuramate released by *L. acidophilus* has an anti-inflammatory effect on LPS-induced inflammation. Decreased N-acetylmuramate in VDR^∆lyz^ mice might be associated with loss of *L. acidophilus* as reported in our previous study^44^. Consistent with the recent report that indicated that high-fat diet depletes the indole-3-carboxylate and other tryptophan derived microbial metabolites, which are known to attenuate weight gain in rats^45^. Because polyamines have strong anti-inflammatory functions^46^, these changes may impact aspects of immunity. Previous studies from our lab have indicated a correlation between the short-chain fatty acid (SCFA) butyrate and VDR^8,15^. Lack of 1,25(OH)2D3 or VDR deficiency results in microbial dysbiosis, leading to greater susceptibility to colitis, which might be important for patients with IBD^8,47–49^. Loss of SCFA and VDR is also connected to a higher risk of colon cancer^50^. Probiotic treatments could potentially exert beneficial effects depending on the VDR status^51^.

Gut microbiota controls neurobehavior via modulating brain insulin sensitivity and metabolism of tryptophan, the precursor of serotonin^52^. Increased influx of tryptophan into the brain by HFD could be related to increased blood insulin levels. VDR^Δlyz^ group displayed significant downregulation of quinolinate and tocopherol pathway derived metabolites and increase in nicotinamide. Quinolinic acid acts as a neurotoxin, proinflammatory mediator, and prooxidant molecule^53^. Loss of intestinal VDR also increased kynurenine, a pathway associated with inflammatory neurological disorder^30^. These changes indicate the role of VDR in neurophysiology. The role of VDR/vitamin D in the gut-brain axis needs further investigation in future research.

We have demonstrated gender differences in metabolites that may be regulated by VDR status. Female mice were shown to be more affected by VDR deletion than male mice. Higher circulating bile acids were observed in obese and type-2 diabetes^54^. Thus, significant elevation of secondary bile acids in VDR deficient females indicates that VDR deficiency plays a critical role in bile acid accumulation. The same increase was not observed in males, suggesting that sex hormones might also play a role in bile acid accumulation. This might be the reason why Vitamin D deficiency makes females more vulnerable to metabolic disorders, including obesity^55^. Polyamines facilitate a switch between different coactivator complexes that bind to nuclear receptors, such as VDR^19^. The increase in polyamines was more significant in male VDR^ΔIEC^ mice, indicating the gender differences in metabolite-VDR interactions.

One of the limitations of our study is that it does not provide information on urolithin and that certain biochemicals did not reach levels of significance. However, we did clearly demonstrate that in the absence of VDR, altered intestinal homeostasis occurred which could drive an altered intestinal metabolism. We need to determine the potential function of the microbiota by functional and taxonomic annotation of the microbiota, using sample-matched data. Other future directions could include plasma samples from these mice, which will further improve our understanding of the role of intestinal homeostasis to host metabolism. As a path forward, we need to validate changes seen in this dataset in various disease models specifically related to metabolic syndrome and in human cohorts (representing both diets and genders).

## Conclusion

VDR is crucial for maintaining a healthy microbiome and metabolome. Our study reports how VDR deficiency not only drives derangements in gut-microbiome but also significantly alters the metabolite profile in a tissue- and gender-specific manner. We have demonstrated the role of nuclear receptors (e.g., VDR and FXR) in regulating host physiology and microbial metabolites in health and obesity. These findings may potentially inform strategies for the prevention and management of metabolic diseases by elevating VDR and restoring host-microbiome-metabolites.

## Methods

### Experimental animals and design

VDR^LoxP^ mice were formerly developed by Dr. Geert Carmeliet. VDR^ΔIEC^ mice were obtained by crossing the VDR^LoxP^ mice with villin-cre mice and VDR^Δlyz^ mice were obtained by crossing Lyz-cre mice. Both Vilin-cre and Lyz-cre mice purchased from Jackson Laboratories. VDR^ΔIEC^ mice were derived from heterozygous mating pairs so that wild type and conditional KO mice came from the same litter. The same breeding method was used for the VDR^LoxP^ mice.

Six-week old VDR^ΔIEC^ (8 female and 9 male), VDR^Δlyz^ (5 female and 5 male), and VDR^LoxP^ (6 female and 10 male) mice were received normal chow (10% fat calories) diet. All mice were housed in specific pathogen-free environments under a controlled condition of 12 h light/12 h dark cycle at 20–22 °C and 45  ±  5% humidity, with free access to food and ultrapure water. All animal work was approved by the University of Illinois at Chicago Committee on Animal Resources. All experiments were performed in accordance with relevant guidelines and regulations.

This was a three-way (Genotype, Diet Treatment, and Gender) study design. To further evaluate the diet effect of VDR^ΔIEC^ versus VDR^LoxP^, an additional 7 VDR^ΔIEC^ (3 female and 4 male) and 6 VDR^LoxP^ (4 female and 2 male) mice were fed with high-fat diet (45% fat calories) for 16 weeks. The body weights and food intake of all animals were observed once a week during the experiments. Fecal contents of mice were carefully collected in separate Eppendorf tubes, labeled with unique identification number and stored at −80 °C until sipped. Samples were transported to Metabolon Inc, NC, USA in dry ice by overnight shipment for analysis.

### Sample preparation

Fecal samples were prepared using the automated MicroLab STAR® system from Hamilton Company. Several recovery standards were added prior to the first step in the extraction process for QC purposes. To remove protein, dissociate small molecules bound to protein or trapped in the precipitated protein matrix, and to recover chemically diverse metabolites, proteins were precipitated with methanol under vigorous shaking for 2 min (Glen Mills GenoGrinder 2000) followed by centrifugation. The resulting extract was divided into five fractions: two for analysis by two separate reverse phase (RP)/UPLC-MS/MS methods with positive ion mode electrospray ionization (ESI), one for analysis by RP/UPLC-MS/MS with negative ion mode ESI, one for analysis by HILIC/UPLC-MS/MS with negative ion mode ESI, and one sample was reserved for backup. Samples were placed briefly on a TurboVap® (Zymark) to remove the organic solvent. The sample extracts were stored overnight under nitrogen before preparation for analysis.

### Western blotting

Colonic mucosa and liver tissues from mice were isolated and sonicated in lysis buffer (1% Triton X-100, 150 mmol/L NaCl, 10 mmol/L Tris pH 7.4, 1 mmol/L EDTA, 1 mmol/L EGTA pH 8.0, 0.2 mmol/L sodium ortho-vanadate, and protease inhibitor cocktail), as previously described^8^. Primary antibodies to mouse VDR, β-actin (Sigma-Aldrich, Milwaukee, WI, USA), and FXR (Santacruz, CA, USA) were used. WB was finally visualized using an ECL kit. The relative abundance of protein was determined using Image-J (NIH) software. The gels/blots used in figures are checked their compliance with the digital image and integrity policies in *Nature* publisher.

### Immunohistochemical staining (IHC)

Immunohistochemistry (IHC) was performed using sections of paraffin-embedded liver tissue as previously described^6^. Sections were incubated in primary antibody FXR (Santacruz, CA, USA) 1:50 diluted for in blocking buffer) at 4 °C overnight. Then washed three times with 0.1% Tween in PBS, and incubated with biotin-conjugated secondary antibody at room temperature for 1 h, washed, incubated with ABC reagent at RT for 1 h (Vector lab PK-6100 standard), washed, visualized with DAB kit (Vector lab SK-4100) and counterstained with hematoxylin. Images were captured by B21 fluorescence microscope.

### Shotgun sequencing

Fecal samples were used for shotgun sequencing. genomic DNA was fragmented into relatively small pieces (generally 250-600 bp fragments) prior to sequencing. Subsequently, the known sequences are used to manipulate the DNA by way of PCR amplification (to increase the total amount of DNA but without selecting for any specific sequences) and for the initiation of the sequencing reaction, again without selection for any specific sequence from the source genomic DNA. Millions to hundreds of millions of short sequences (generally 150 bases, in pairs) are generated using Illumina sequencing platforms. These data were analyzed by (a) searching for lineage-specific marker genes; (b) high-throughput BLAST analysis of individual sequences against a reference sequence; (c) assembly of larger DNA sequences (“contigs”) from the short-read data. Ultimately, the annotated data can be used to characterize the gene content of microbial communities, measure diversity, and identify differences in the relative abundance of microbial features (i.e., taxa, genes, and pathways) between different groups of samples. We used the UIC genomic facility for our studies.

### Statistical analysis

Raw data were extracted, peak-identified and QC processed using Metabolon’s hardware and software. Compounds were identified by comparison to library entries of purified standards or recurrent unknown entities. Furthermore, biochemical identifications are based on three standard criteria. A variety of procedures were carried out to ensure that a high-quality data set was made available for statistical analysis and data interpretation. Metabolites were quantified and data were normalized as necessary.

The analysis data were presented as a fold change ratio of treatment vs. control. All the tests were two-sided. The numbers of biochemicals were summarized as statistical significance at P ≤ 0.05 and 0.05 < P < 0.10 levels. Following log transformation and imputation of missing values as appropriate, a two-way ANOVA with contrasts and Welch’s two-sample *t*-test were used to identify biochemicals that differed significantly between genotypes and treatment groups. Three-way ANOVA was further conducted to identify biochemicals exhibiting significant interaction and main effects for experimental parameters of genotype, diet, and gender. An estimate of the false discovery rate (*q*-value) was calculated to take into account the multiple comparisons that normally occur in metabolomic-based studies. The matched pairs *t*-test is used to test whether two unknown means are different from paired observations taken on the same sample. To present the high-level overview of data structure for experimental parameters of genotype, diet, and gender, principal component analysis (PCA) along with hierarchical clustering analysis (HCA) as well as random forest (RF) analysis were conducted to highlight biochemical alterations between fecal samples collected from VDR^ΔIEC^, VDR^∆lyz^, and VDR ^LoxP^, mice that were kept either on HFD or chow diet as well as gender differences.

### Ethics approval and consent to participate

No human study. All animal studies were performed following ACC guidelines at the University of Illinois at Chicago (UIC), IL, USA.

## Supplementary information

Supplement figure.

## Data Availability

Data and material will be available by request.
