# Supplementary material for: Vitamin D receptor promotes healthy microbial metabolites and microbiome
Source: Sci Rep. 2020 Apr 30;10:7340. doi: 10.1038/s41598-020-64226-7 (PMC7192915; doi:10.1038/s41598-020-64226-7)
Supplement: Supplementary file 1 — Supplement figure. [file 41598_2020_64226_MOESM1_ESM.pdf]

Vitamin D receptor promotes healthy microbial metabolites and microbiome

Ishita Chatterjee[1], Rong Lu[1], Yongguo Zhang[1], Jilei Zhang[1], Yang Dai[2], Yinglin Xia[1], Jun Sun[1]

1. Division of Gastroenterology and Hepatology, Department of Medicine, University of Illinois at Chicago
2. Bioengineering Department, University of Illinois at Chicago

Running title: Loss of VDR alters microbial metabolites.

Corresponding Authors

Jun Sun, PhD, AGA Fellow, Professor

Division of Gastroenterology and Hepatology, Department of Medicine,

University of Illinois at Chicago,

840 S Wood Street,

Room 704 CSB, MC716,

Chicago, IL, 60612, USA

Tel: 312-996-5020,

E-mail: junsun7@uic.edu

Yinglin Xia, PhD, Research Associate Professor

Department of Medicine, University of Illinois at Chicago

840 S Wood Street,

Room 734 CSB, MC716,

Chicago, IL 60612, USA

Tel: 312-355-4833

E-mail: yxia@uic.edu

Supplementary Figure 1: PCA assessing all fecal samples show significant separation based on the (A) diet and (B) gender, each group is indicated by specific colors as mentioned in the inset. Arrow indicates the shift of group.

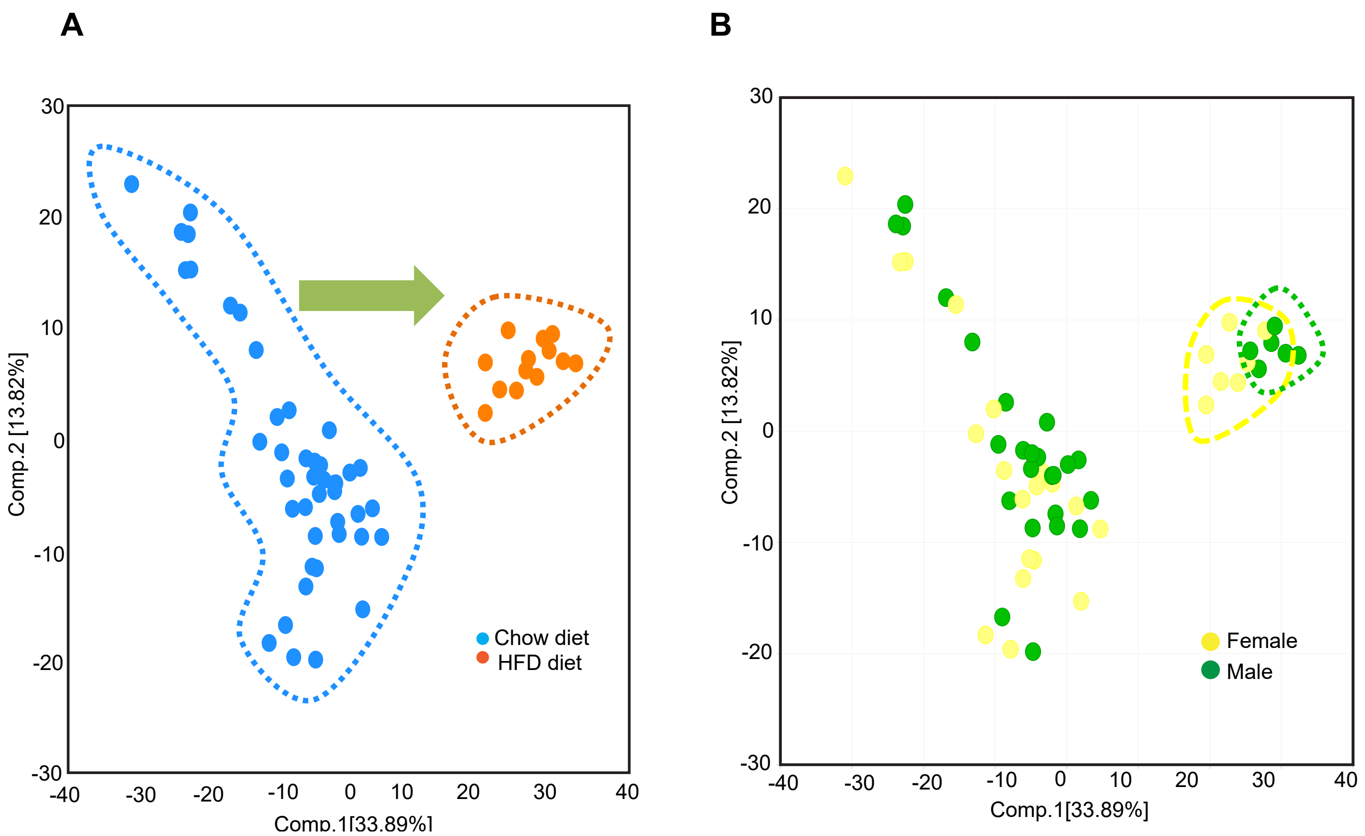

Original Images of western blots for Fig. 10

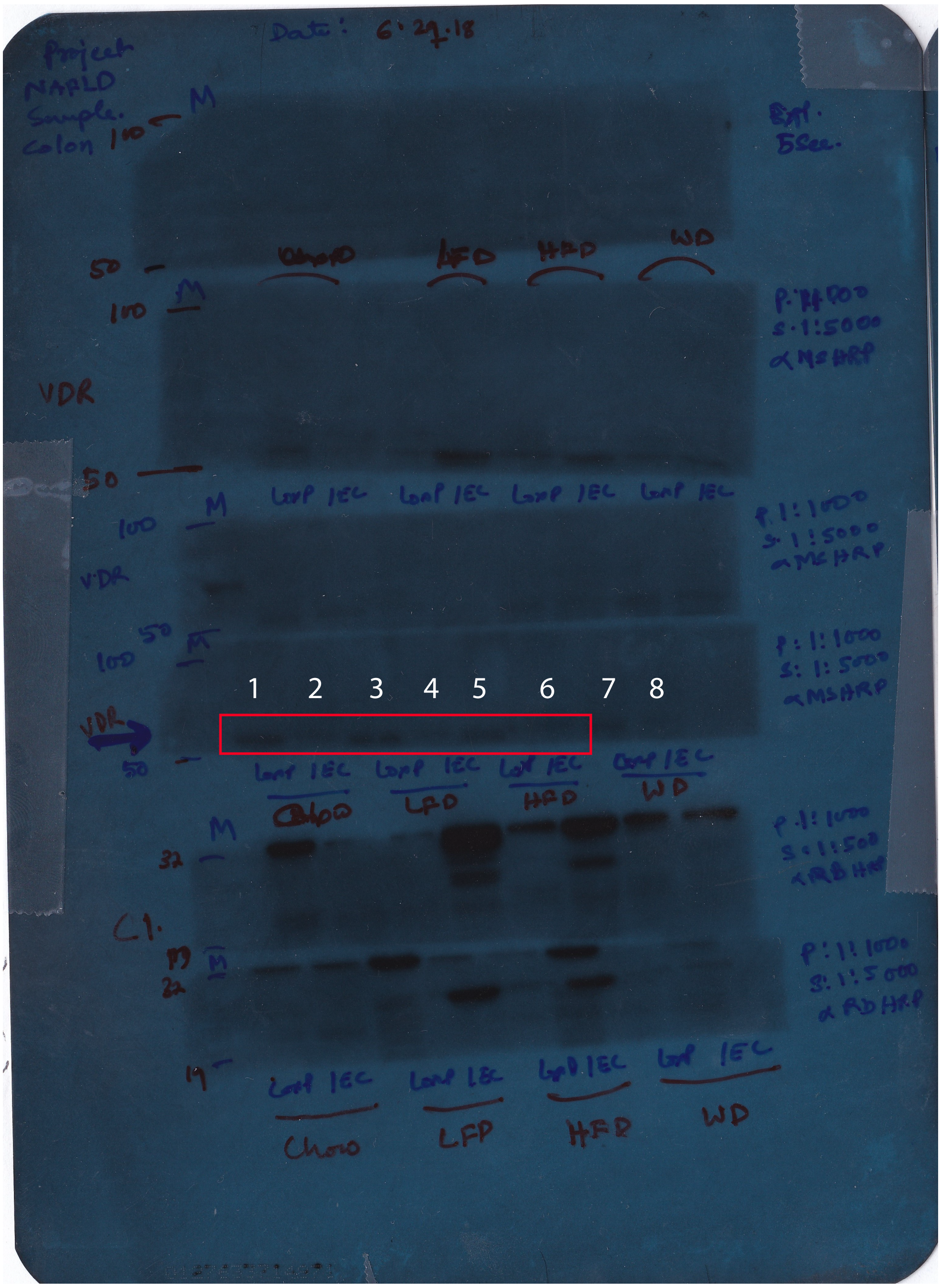

Project NAFLD
Sample. colon
Date: 6.29.18
M
50
100
VDR
LFD
HFD
WD
1 2 3 4 5 6 7 8
32
Chow
LFD
HFD
WD

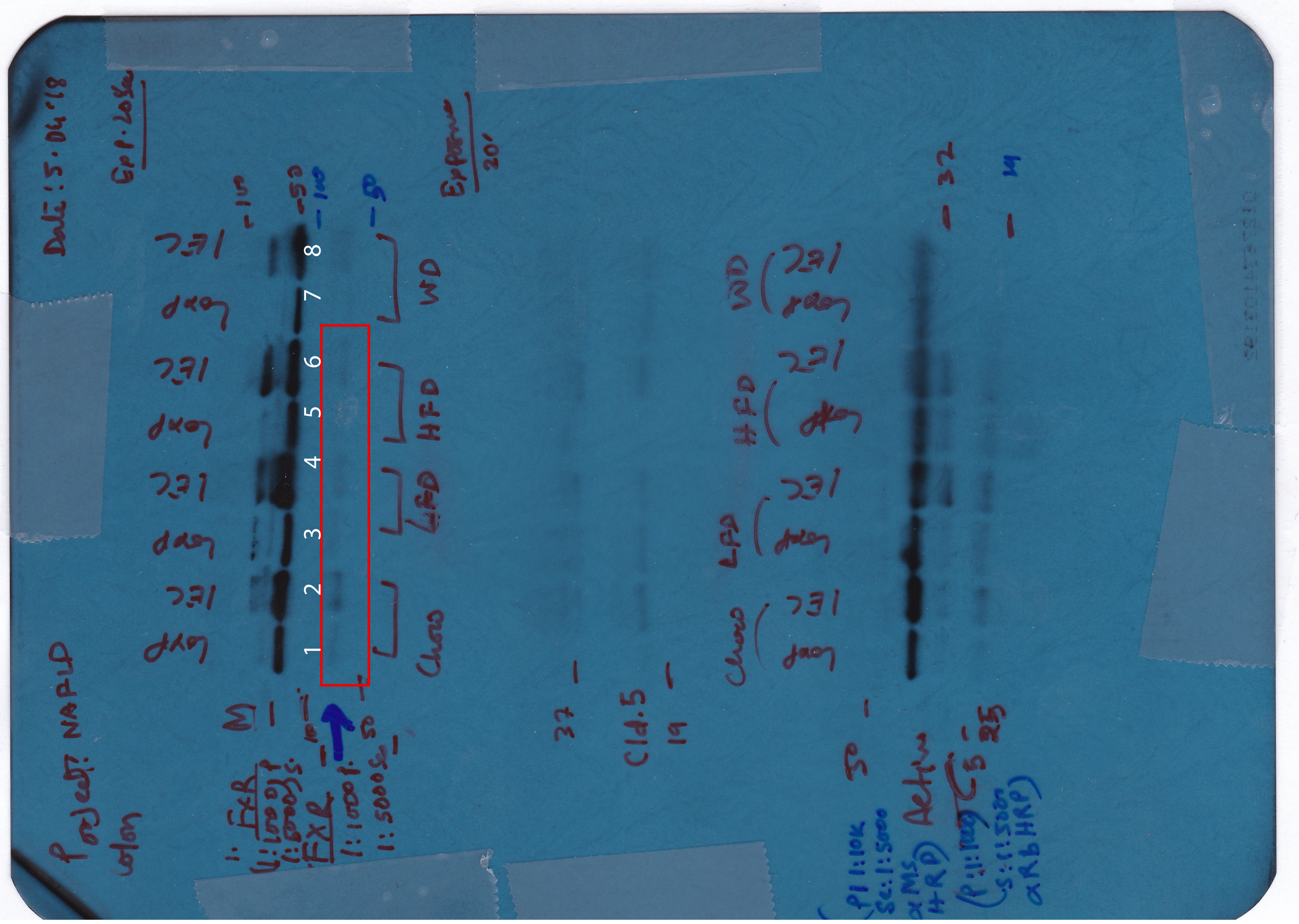

Project: NAFLD
Date: 5.04.18
1 2 3 4 5 6 7 8
Chow
LFD
HFD
WD
Actin

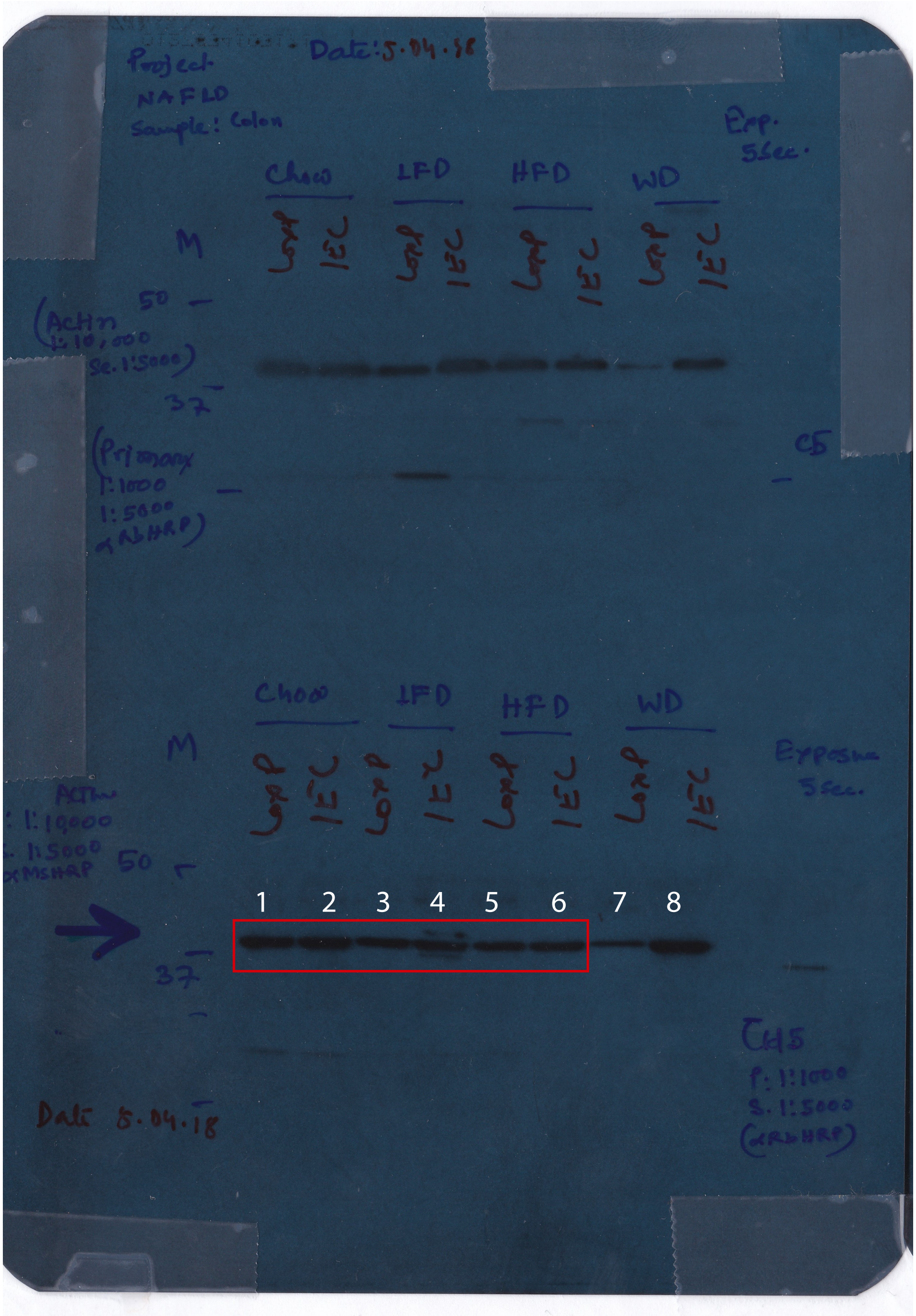
Project
NAFLD
Sample: Colon
Date: 5.04.18
Exp. 5sec.
Chow
LFD
HFD
WD
M
LoxP
IEC
50
37
Actin
1:10,000
Se. 1:5000
Primary
1:1000
1:5000
αRbHRP
C5
Exposure
5sec.
1
2
3
4
5
6
7
8
CH5
P: 1:1000
S. 1:5000
(αRbHRP)
Date 8.04.18

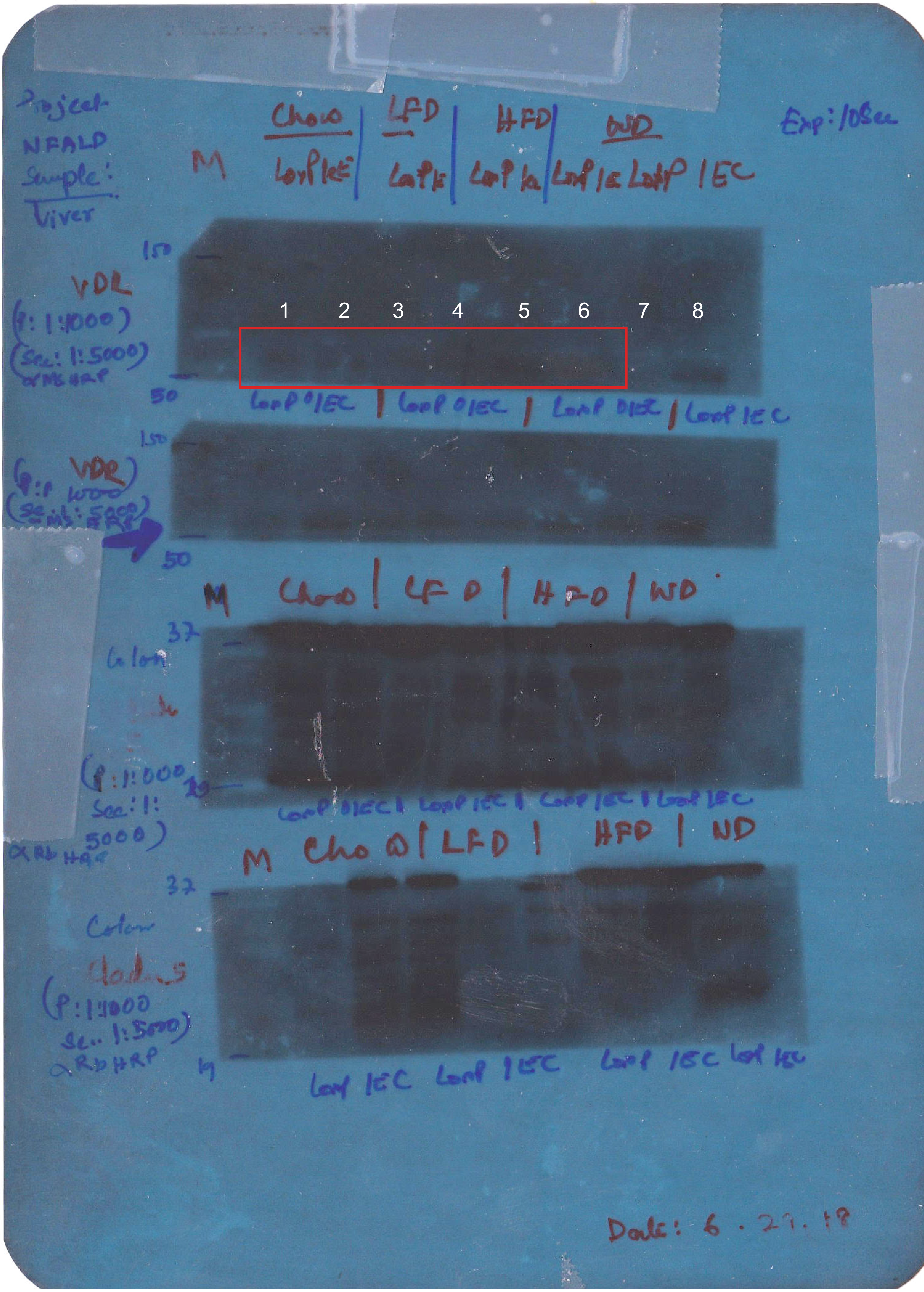
Project
NFALD
Sample:
Liver
Chow
LFD
HFD
WD
Exp: 10Sec
M
VDR
1 2 3 4 5 6 7 8
150
50
M Chow | LFD | HFD | WD
37
Colon
(P: 1:1000
Sec: 1:
5000)
M Chow | LFD | HFD | WD
37
Colon
(P: 1:1000
Sec: 1:5000)
αRb HRP
Date: 6.27.18

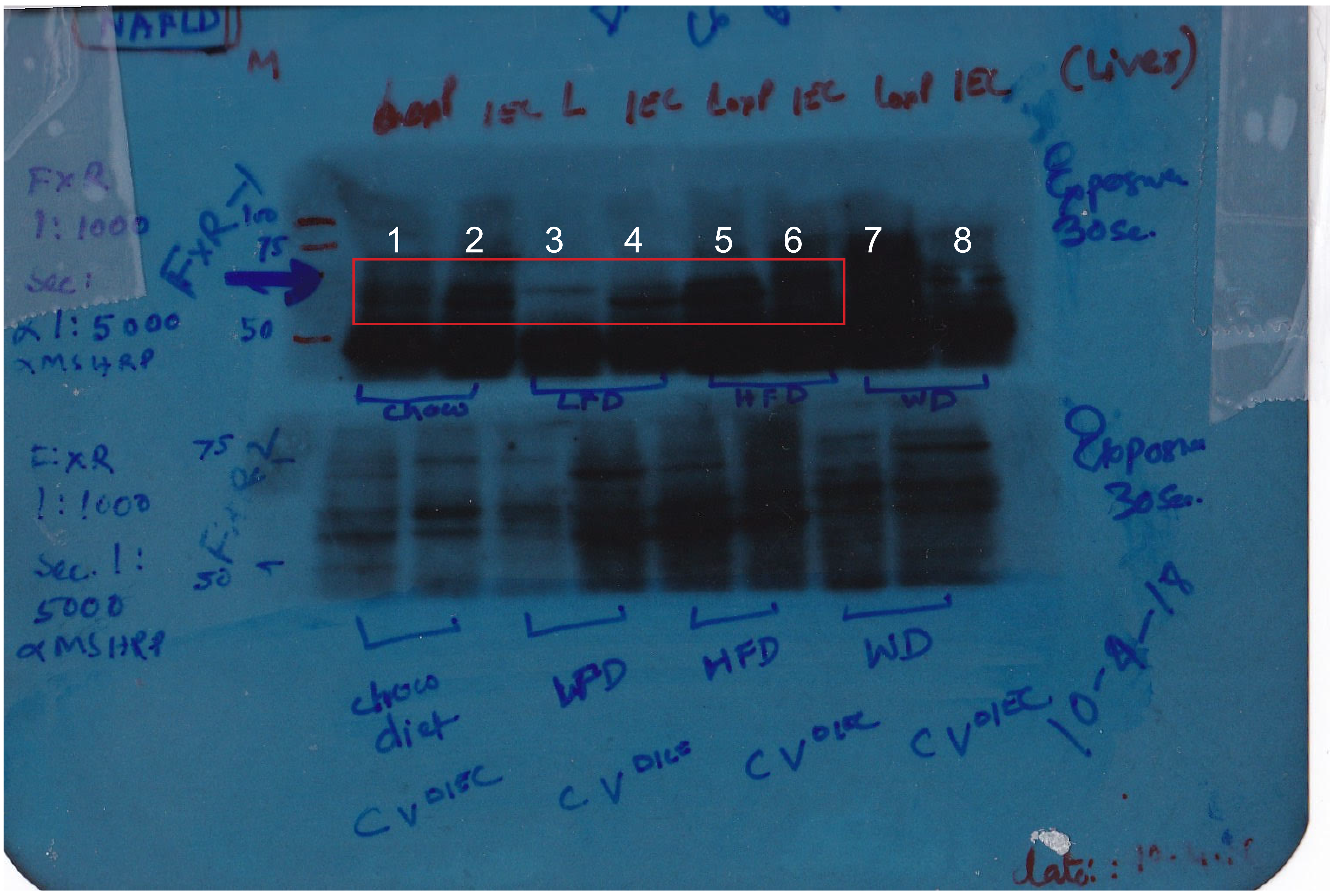

NAFLD
M
(Liver)
FxR
1:1000
Sec:
α 1:5000
α MS HRP
FXR
100
75
50
1 2 3 4 5 6 7 8
Chow
LFD
HFD
WD
Exposure
30sec.
75
50
1:1000
Sec. 1:
5000
α MS HRP
chow
diet
LFD
HFD
WD
CV
CV
CV
CV
10-4-18

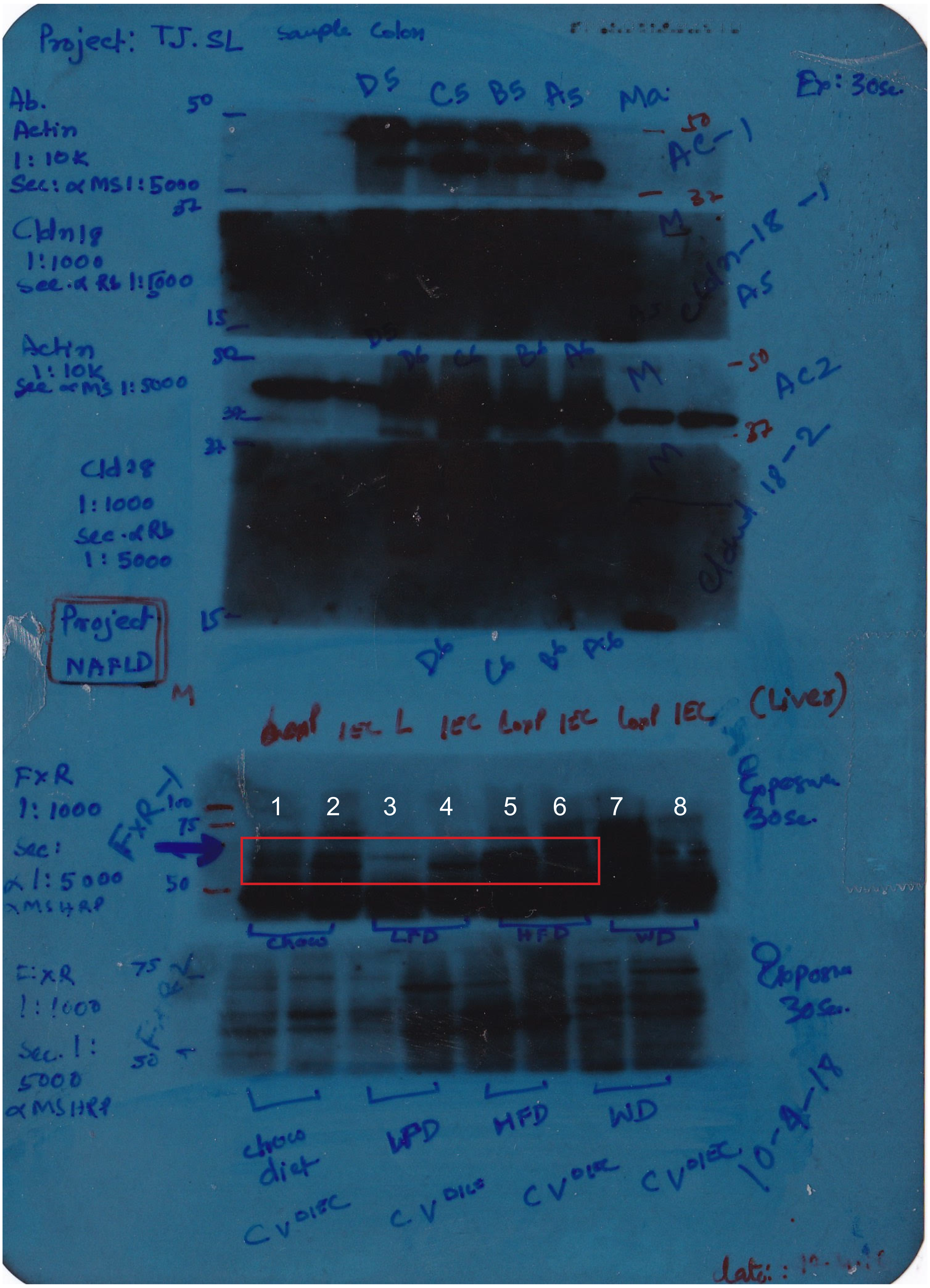

Project: TJ.SL
sample Colon
Ab.
Actin
1:10K
Sec: αMS 1:5000
Cldn18
1:1000
sec·α Rb 1:5000
Actin
1:10K
Sec αMS 1:5000
Cld18
1:1000
Sec·α Rb
1:5000
Project
NAFLD
(Liver)
FxR
1:1000
Sec:
α 1:5000
αMS HRP
1 2 3 4 5 6 7 8
Chow
HFD
WD
Exposure
30sec.
FxR
1:1000
Sec. 1:
5000
αMS HRP
chow diet
HFD
WD
10-4-18

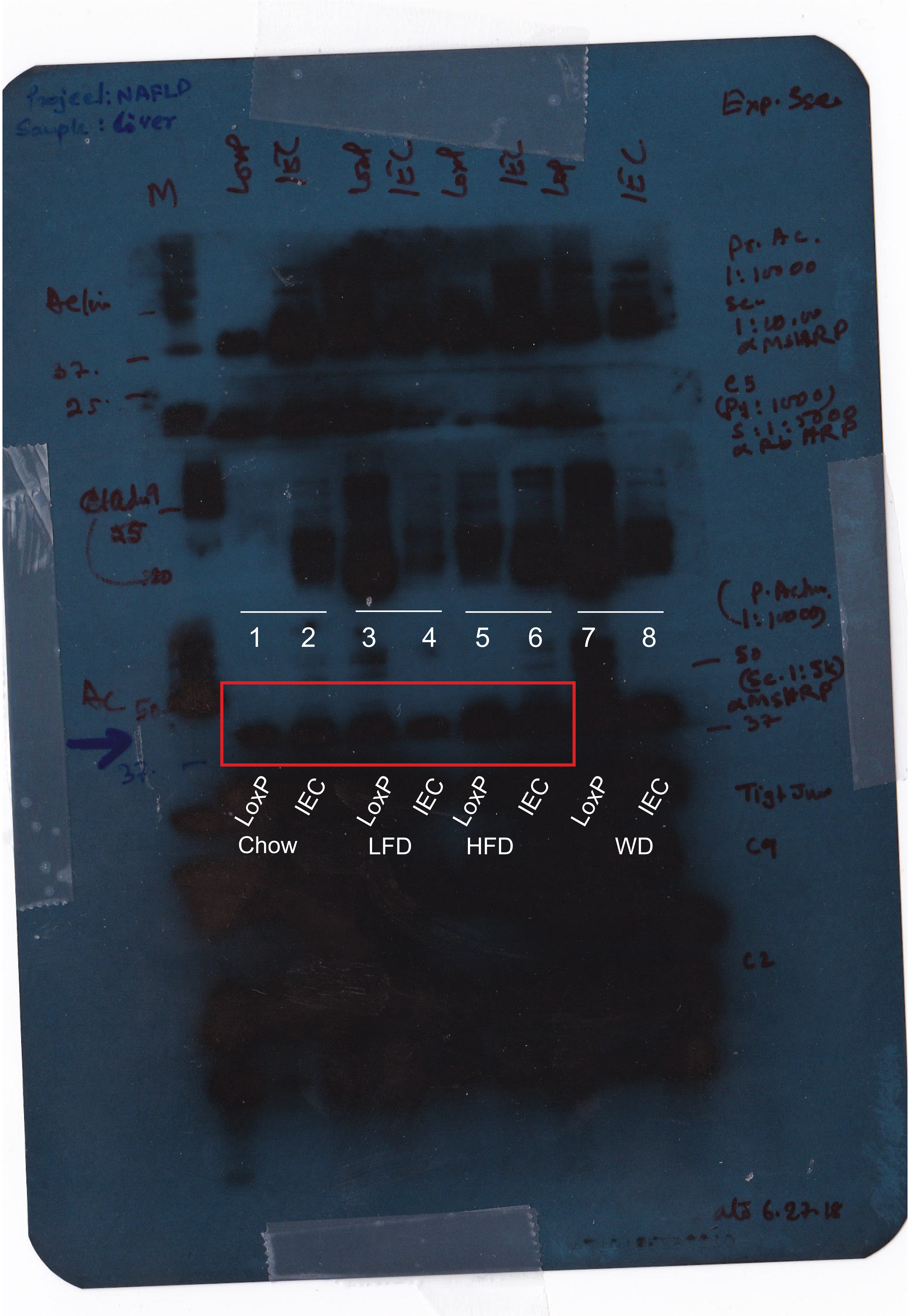
Project: NAFLD
Sample: Liver
Exp. 5sec
M
1 2 3 4 5 6 7 8
LoxP IEC LoxP IEC LoxP IEC LoxP IEC
Chow
LFD
HFD
WD
Tight Junc
C2

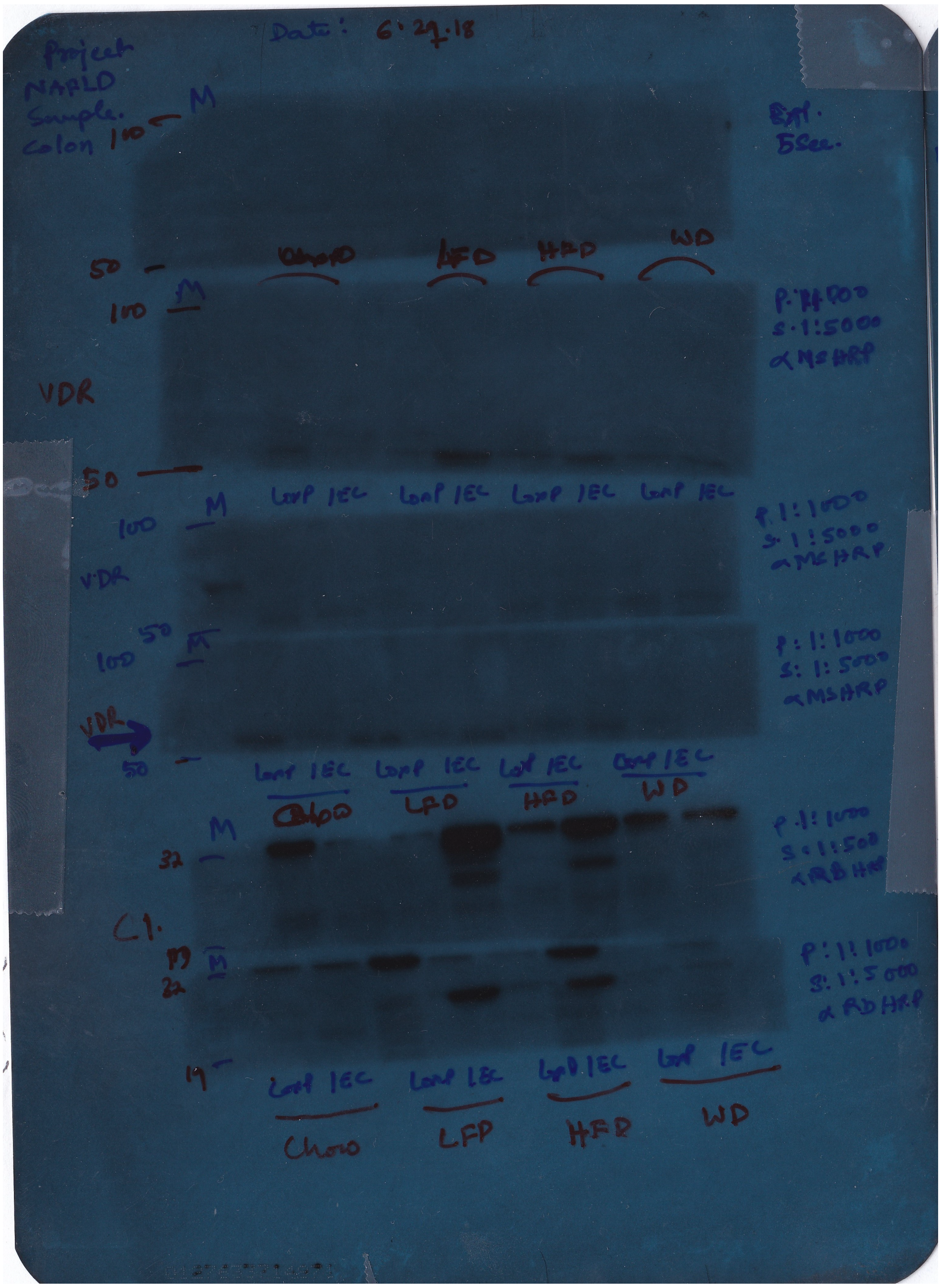
Project
NAFLD
Sample.
colon
Date: 6.29.18
M
VDR
WD
Lamp IEC
Chow
LFD
HFD
WD
P: 1:1000
S: 1:5000
P: 1:1000
S: 1:5000

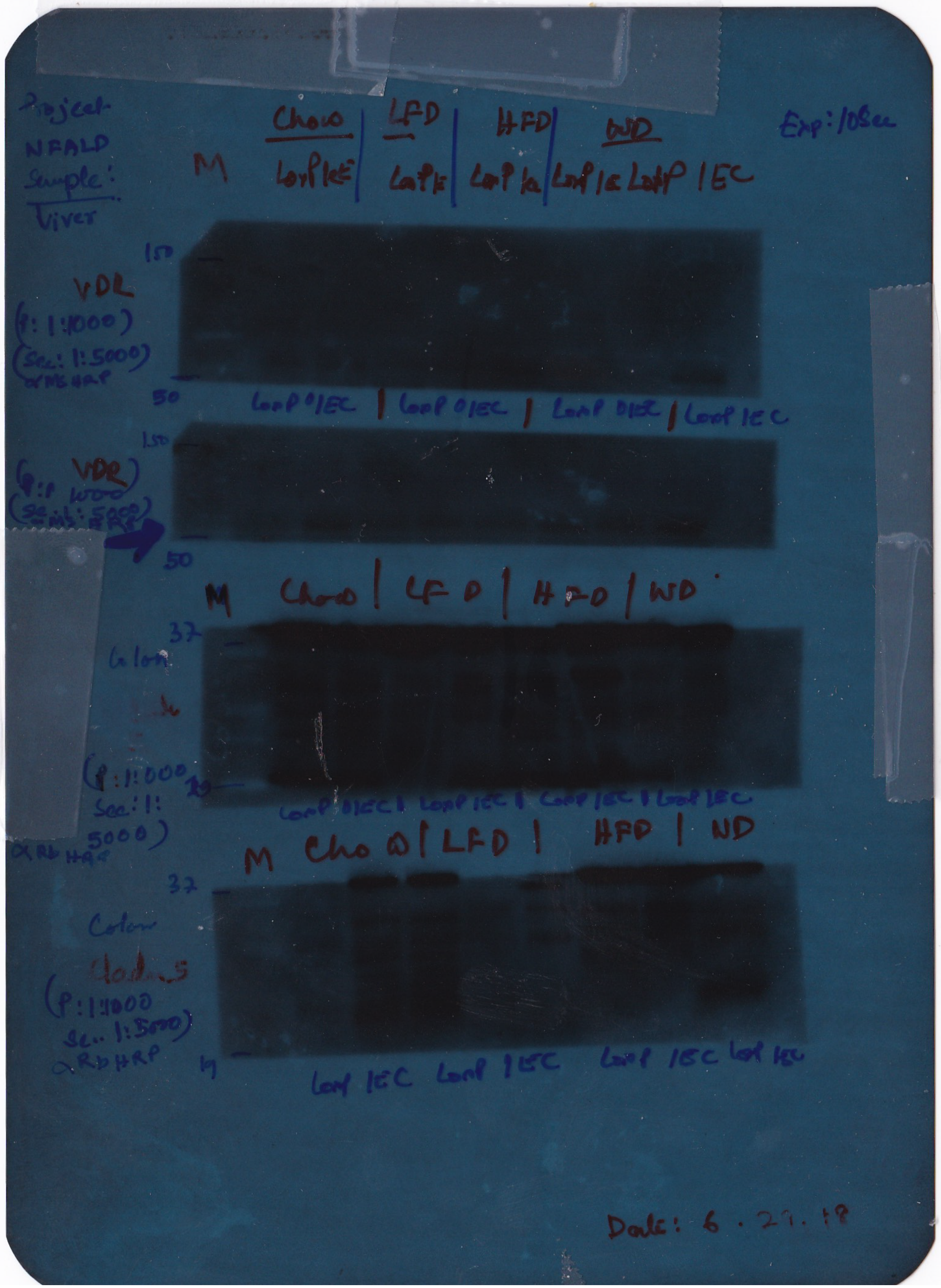
Project
NFALD
Sample:
Liver
M
Chow
LFD
HFD
WD
Exp: 10sec
150
50
VDR
M Chow | LFD | HFD | WD
37
20
Colon
M Chow | LFD | HFD | WD
37
Colon
Date: 6 . 27 . 18

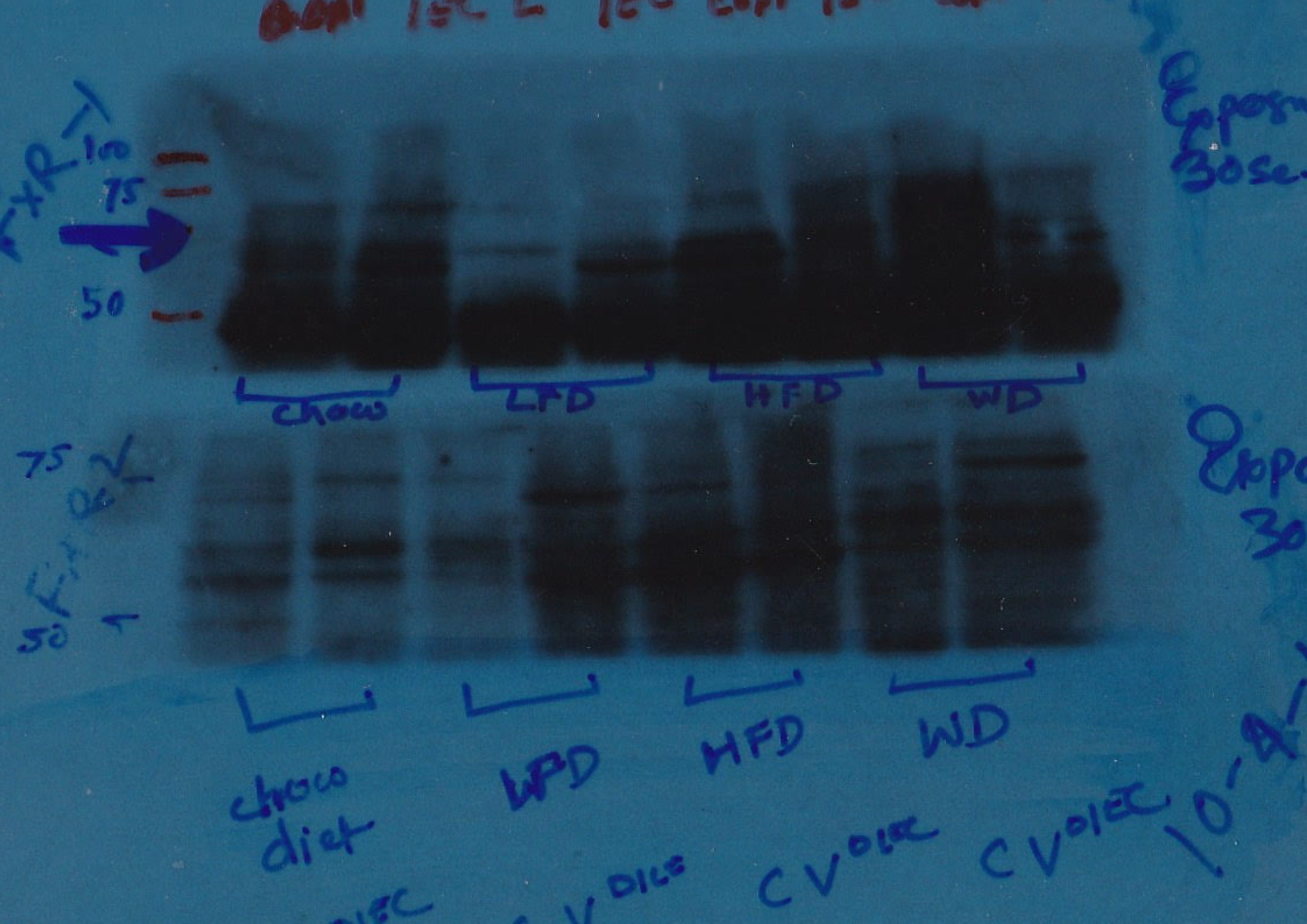

100
75
50
Chow
LFD
HFD
WD
75
50
HFD
WD
diet

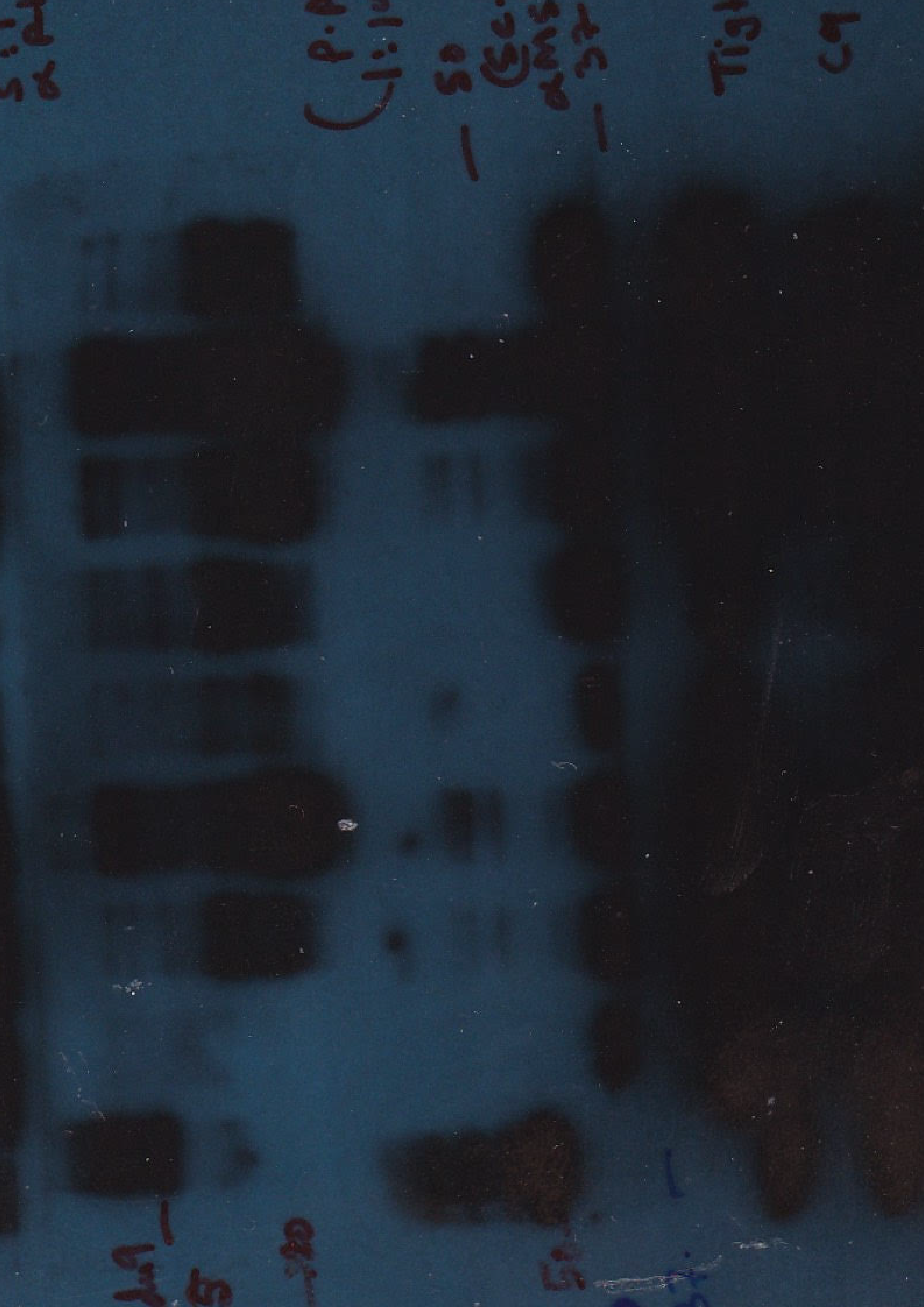

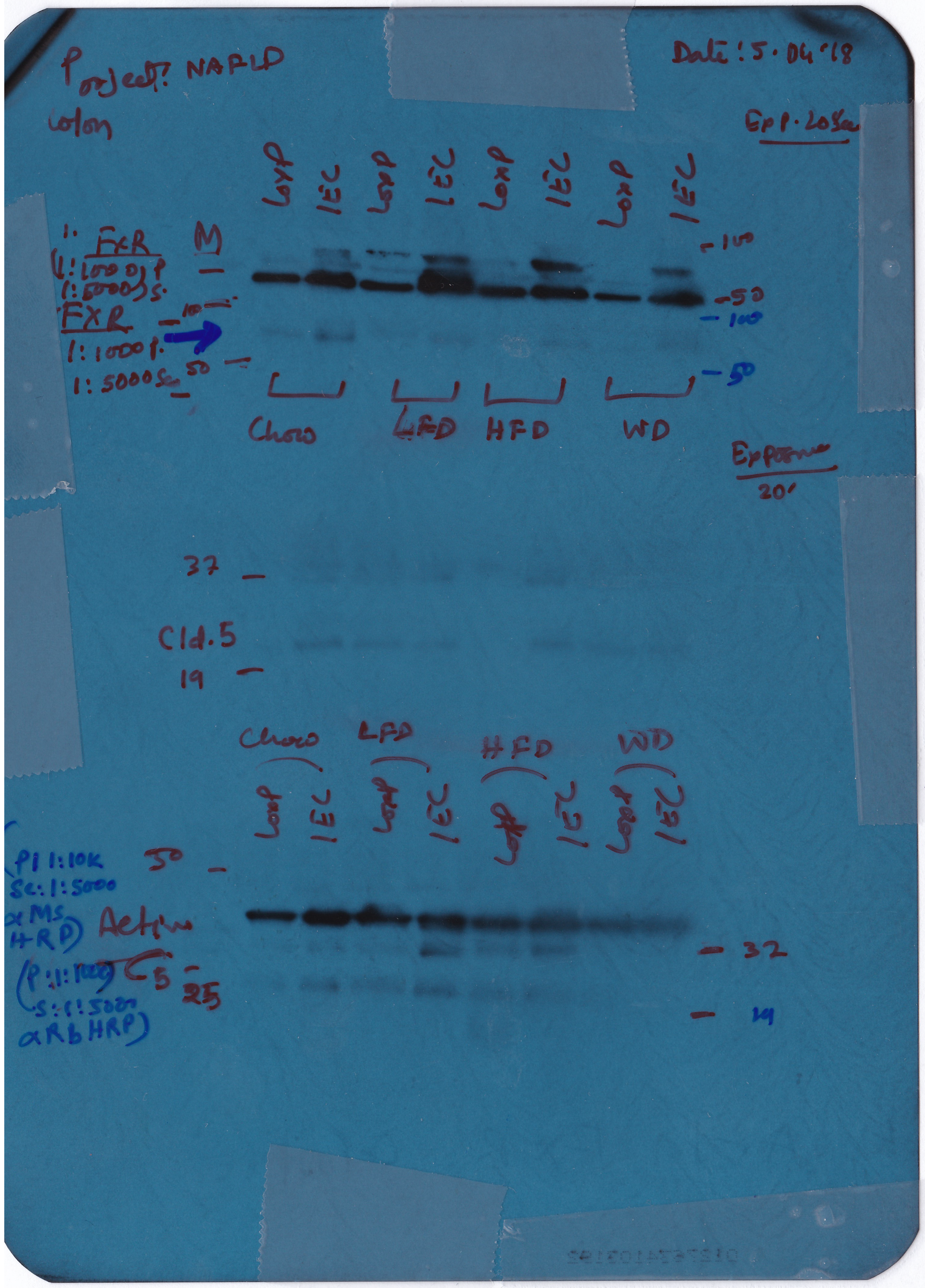
Project: NAFLD
colon
Date: 5.04.'18
Exp. 20Sec
M
1: FXR
1:1000 P
1:5000 S.
FXR
1:1000 P.
1:5000 S.
LoxP
IEC
LoxP
IEC
LoxP
IEC
LoxP
IEC
100
50
100
50
Chow
LFD
HFD
WD
Exposure
20'
37
Cld.5
19
Chow
LFD
HFD
WD
LoxP
IEC
LoxP
IEC
LoxP
IEC
LoxP
IEC
P1 1:10K
Se: 1:5000
αMS HRP
Actin
50
32
19
25
αRb HRP)

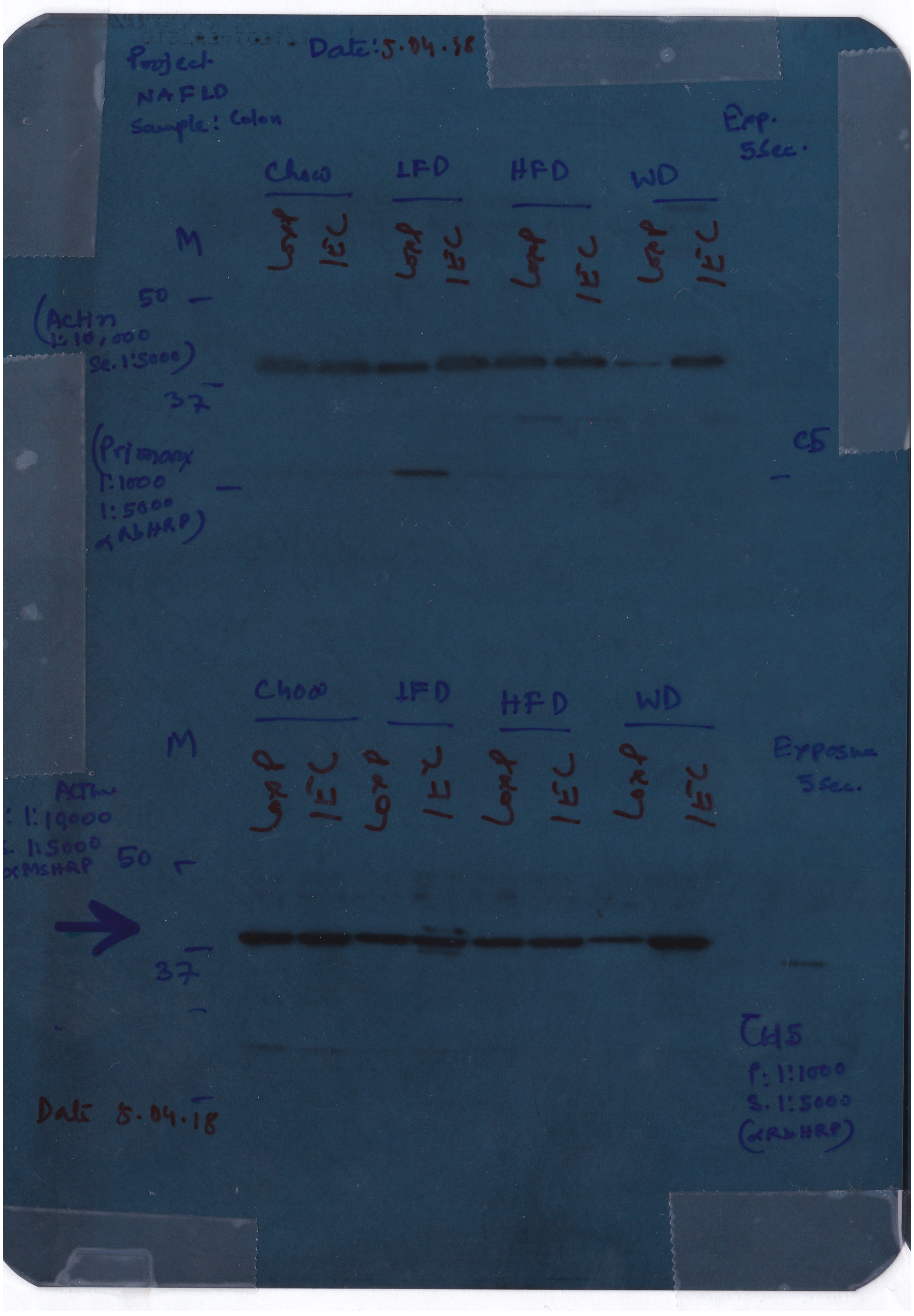
Project
NAFLD
Sample: Colon
Exp.
5sec.
Chow
LFD
HFD
WD
M
50
37
Exposure
5sec.
50
37
P: 1:1000
S. 1:5000
Date 8.04.18
